# A diketopyrrolopyrrole dye-based dyad on a porous TiO_2_ photoanode for solar-driven water oxidation†

**DOI:** 10.1039/d0sc04509h

**Published:** 2020-09-25

**Authors:** Daniel Antón-García, Julien Warnan, Erwin Reisner

**Affiliations:** Department of Chemistry, University of Cambridge Lensfield Road Cambridge CB2 1EW UK reisner@ch.cam.ac.uk

## Abstract

Dye-sensitised photoanodes modified with a water oxidation catalyst allow for solar-driven O_2_ evolution in photoelectrochemical cells. However, organic chromophores are generally considered unsuitable to drive the thermodynamically demanding water oxidation reaction, mainly due to their lack of stability upon photoexcitation. Here, the synthesis of a dyad photocatalyst (**DPP-Ru**) consisting of a diketopyrrolopyrrole chromophore (**DPPdye**) and ruthenium-based water oxidation catalyst (**RuWOC**) is described. The **DPP-Ru** dyad features a cyanoacrylic acid anchoring group for immobilisation on metal oxides, strong absorption in the visible region of the electromagnetic spectrum, and photoinduced hole transfer from the dye to the catalyst unit. Immobilisation of the dyad on a mesoporous TiO_2_ scaffold was optimised, including the use of a TiCl_4_ pretreatment method as well as employing chenodeoxycholic acid as a co-adsorbent, and the assembled dyad-sensitised photoanode achieved O_2_ evolution using visible light (100 mW cm^−2^, AM 1.5G, *λ* > 420 nm). An initial photocurrent of 140 μA cm^−2^ was generated in aqueous electrolyte solution (pH 5.6) under an applied potential of +0.2 V *vs.* NHE. The production of O_2_ has been confirmed by controlled potential electrolysis with a faradaic efficiency of 44%. This study demonstrates that metal-free dyes are suitable light absorbers in dyadic systems for the assembly of water oxidising photoanodes.

## Abstract 2

Construction of a water oxidising photoanode with an organic chromophore-catalyst dyad.

## Graphical abstract

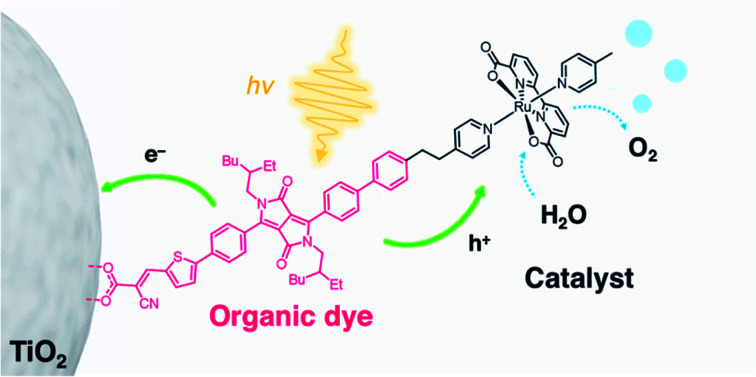

## Introduction

The integration of a molecular dye and a water oxidation catalyst (WOC) onto an n-type metal oxide (*e.g.*, titanium dioxide, TiO_2_) semiconductor (SC) film on a conductive substrate (*e.g.*, fluorine-doped tin oxide, FTO), produces a dye-sensitised photoanode for visible light-driven O_2_ evolution in photoelectrochemical (PEC) cells.^1,2^ Dye-sensitised photoanodes operate by photoexcitation of the dye (S), which results in ultra-fast (typically sub-ns) electron injection from the excited state (S*) to the conduction band (CB) of the semiconductor.^3^ This step is followed by hole transfer to the WOC, which regenerates the oxidised dye (S^+^). Repeated cycles allow the catalyst to accumulate four holes to oxidise water to O_2_.^4–6^ Ruthenium complexes are the most commonly employed molecular WOCs due to their fast O_2_ evolution rates at low overpotentials, with [Ru^II^(bda)(pic)_2_] (bda = 2,2′-bipyridine-6,6′-dicarboxylic acid, pic = 4-picoline) displaying benchmark performance.^7,8^

**Fig. 1:**
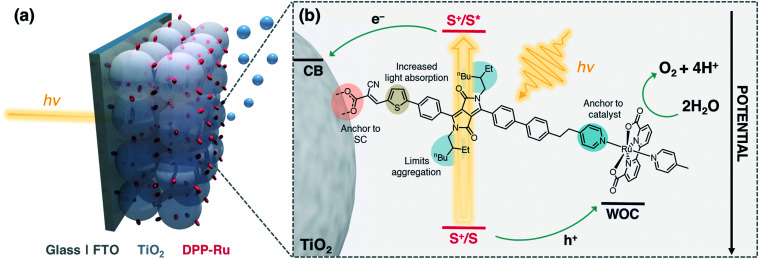
(a) Schematic representation of the water oxidising dyad-sensitised photoanode. (b) Structure of **DPP-Ru** with design features and electron transfer events.

Co-immobilisation of the dye and WOC on an electrode results in fast electron–hole recombination at the molecule–electrode interface, thereby resulting in limited efficiency.^6,9^ These unfavourable recombination dynamics can in principle be overcome by covalently linking the chromophore to the catalyst, forming a dyadic system in which the catalyst is placed farther away from the electrode surface (Fig. 1). Dyads thereby enable fast interfacial electron transfer from the dye to semiconductor electrode, and intramolecular quenching of S^+^ by the WOC combined with slow recombination of semiconductor electrons with holes accumulated in the oxidised WOC.^3,10^

Dyads have previously been constructed using Ru-based dyes and [Ru^II^(bda)] WOCs, and have been integrated in TiO_2_ photoanodes either by immobilisation of the synthesised assembly,^11^ or by *in situ* polymerisation.^12–14^ However, dyad photoanodes typically rely on precious-metal chromophores, and the need for reduced cost has led to the exploration of earth-abundant chromophores with more intense visible light absorption, embodied by metal porphyrins^15^ and organic dyes.^16^ An example of a zinc porphyrin-based dyad has been reported, but the chromophoric unit lacks sufficient oxidation potential for light-activation of the [Ru(bda)]-based WOC.^15^ Organic dyes, frequently designed with push–pull architectures, have been co-immobilised with [Ru^II^(bda)]-type catalysts on dye-sensitised photoanodes, albeit with low efficiencies.^17–23^

Diketopyrrolopyrroles (DPPs) are a class of chromophores known for their high photostability and intense light absorption, which can be tuned to absorb even red and infra-red photons.^24,25^ Immobilisation onto metal oxides in both n-type and p-type dye-sensitised solar cells (DSSCs) has been achieved using a surface anchoring group.^26–31^ Recently, DPPs were also co-immobilised with molecular catalysts on colloidal TiO_2_ nanoparticles, and CuCrO_2_ delafossite and NiO photocathodes for visible light-driven H_2_ evolution.^32,33^

In this study, we report the synthesis, optical properties, electrochemistry and PEC performance of a bespoke molecular dyad, **DPP-Ru** (Fig. 1), where a tailor-made DPP chromophore is covalently linked to a [Ru^II^(bda)]-type WOC. The chromophore unit contains a pyridine moiety to coordinate to the ruthenium catalyst, and an alkyl spacer chain to provide flexibility for the dimerisation of the Ru unit for improved catalysis.^11,34–36^ A thiophene unit increases planarity and shifts the absorption maximum to longer wavelengths, allowing more incident solar photons to be harvested.^37,38^ The incorporation of bulky alkyl chains on the DPP core allow for increased hydrophobicity and limit π–π aggregation of the molecule.^25^**DPP-Ru** is then immobilised on a porous TiO_2_ electrode *via* a cyanoacrylic acid anchoring group, which allows for localisation of the LUMO near the electrode surface, facilitating electron injection into the bulk of the TiO_2_ semiconductor.^30^ Optimisation of immobilisation conditions and surface coverage results in a dyad-sensitised photoanode for light-driven water oxidation to O_2_.

## Results and discussion

### Synthesis and characterisation

**Scheme 1:**
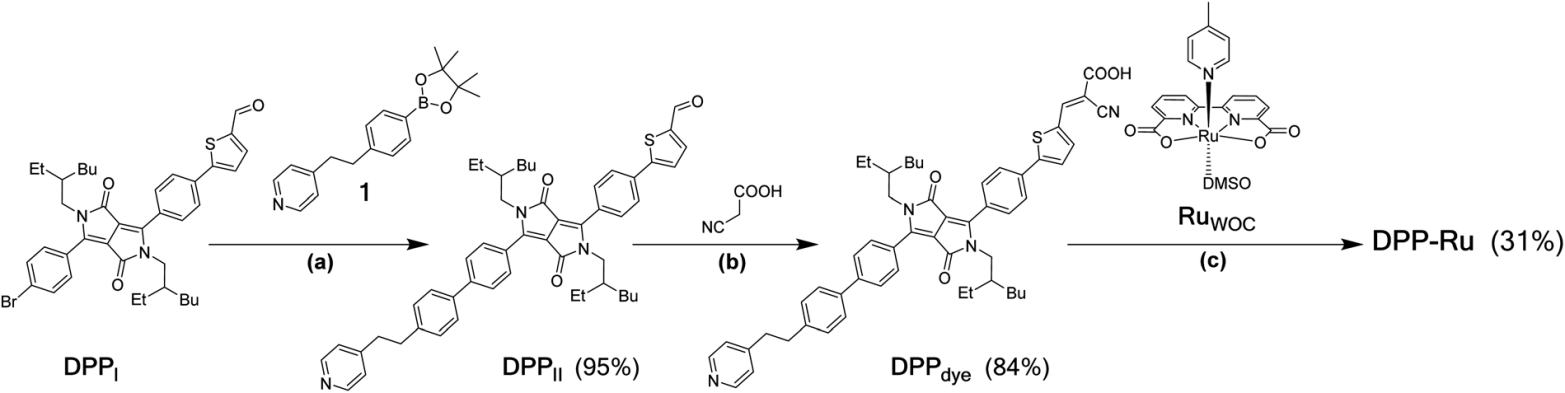
Synthesis of the water oxidising **DPP-Ru** dyad. Conditions: (a) [Pd(PPh_3_)_4_], Na_2_CO_3_, tetrahydrofuran/H_2_O 2 : 1, 87 °C, N_2_, overnight; (b) piperidine, tetrahydrofuran, reflux, N_2_, overnight; (c) MeOH, drops of triethylamine, reflux, N_2_, overnight.

The **DPP-Ru** dyad (Fig. 1b) was prepared by a convergent synthesis protocol, firstly forming the bespoke chromophore, **DPPdye**, followed by complexation to the ruthenium moiety, **RuWOC** [Ru^II^(bda)(dmso)(pic)] (dmso = dimethyl sulfoxide) (Scheme 1). The synthesis of **DPPdye** started by preparation of the bridging moiety, **1**, through a two-step synthesis from 4-picoline (Scheme S1†). A Suzuki cross-coupling with **DPPI** (synthesised by a previously reported procedure)^39^ afforded **DPPII** with a yield of 95%. A Knoevenagel condensation with cyanoacetic acid in the presence of piperidine afforded **DPPdye** in 84% yield. Finally, reflux of **RuWOC** (synthesised following a previously reported procedure)^40^ with **DPPdye** in methanol (MeOH) and triethylamine afforded the dye-catalyst assembly **DPP-Ru** in 31% yield. The crude product contained a mixture of **DPPdye**, **DPP-Ru**, [Ru^II^(bda)(pic)_2_] and [Ru^II^(bda)(**DPPdye**)_2_], and accounts for the lower yield. The compounds were purified by column chromatography, and the composition and purity were confirmed by ^1^H, ^13^C and ^11^B NMR spectroscopy (Fig. S1–S5†), high-resolution mass spectrometry, infrared spectroscopy and elemental analysis (see ESI for details†).

### Photophysical properties

**Fig. 2:**
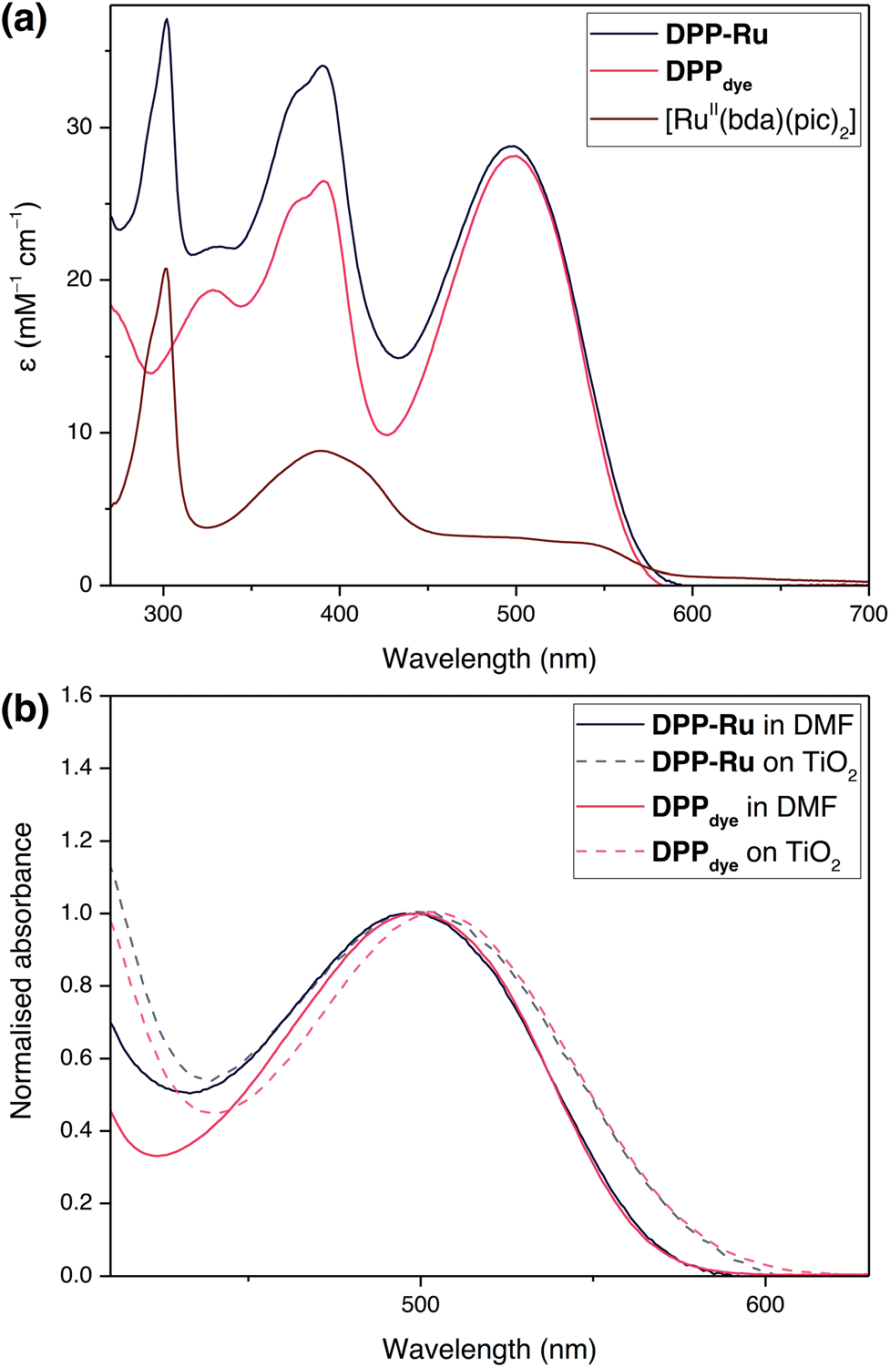
(a) UV-vis absorption spectra of **DPPdye**, [Ru^II^(bda)(pic)_2_] and **DPP-Ru** in DMF solution. (b) Normalised UV-vis spectra of **DPPdye** and **DPP-Ru** on a mTiO_2_ film coated on a glass slide, and in DMF solution.

The UV-vis absorption spectra of **DPPdye**, **DPP-Ru** and [Ru^II^(bda)(pic)_2_] were recorded in *N*,*N*-dimethylformamide (DMF) solution (Fig. 2a). The spectrum of **DPPdye** features an intense band at 499 nm (*ε* = 27.9 mM^−1^ cm^−1^), matching the highest intensity of the solar spectrum, and a tailing absorption up to 560 nm. This characteristic DPP-absorption is attributed to a π–π* HOMO–LUMO transition. Density functional theory (DFT) calculations on similar DPP chromophores have indicated that this transition originates from the DPP core and extends to the cyanoacrylic acid group.^39^ The second band at 390 nm can be attributed to a HOMO−1 to LUMO and HOMO to LUMO+1 transition.^39^

Due to the break in conjugation between the dye and the catalyst, introduced by the ethylene-bridge, only a marginal change in the DPP absorption spectrum is observed upon complexation with the Ru centre in **DPP-Ru**. The dyad also features a strong absorption band at 302 nm, also observed in the [Ru^II^(bda)(pic)_2_] spectrum, which is characteristic of [Ru^II^(bda)] complexes.^41^

Upon photoexcitation at 499 nm, the emission spectrum of **DPPdye** shows a broad band centred at 587 nm, with a vibronic shoulder at 627 nm, in aerated DMF solution (Fig. S6†). The large Stokes shift is typical of phenyl flanked DPP dyes, due to their torsion angle.^42^ The emission trace is similar for **DPP-Ru** (Fig. S7†). Absolute quantum yield measurements for **DPPdye** reached 65%, whereas **DPP-Ru** achieved 4%. This efficient luminescence quenching is indicative of an intramolecular electron transfer from the ruthenium centre to the excited chromophoric unit.^43^

Immersion of a mesoporous TiO_2_ film (anatase nanoparticles, *ca.* 20 nm diameter, *ca.* 6 μm thick) coated on a glass slide in a solution of **DPPdye** and **DPP-Ru** in dichloromethane (DCM) leads to a strong colouration of the electrodes, demonstrating the affinity of the molecules to the metal oxide surface.^44^ The spectra also remain largely unchanged upon immobilisation, confirming that the molecules retain their absorption properties on the electrode (Fig. 2b).

### Electrochemical properties

Using cyclic voltammetry (CV), the electrochemical properties of **DPPdye** and **DPP-Ru** were examined in solution (DMF) and when chemisorbed on a mesoporous indium tin oxide (mITO; particle size < 50 nm, film thickness ∼3 μm)^45–47^ electrode in acetonitrile (MeCN), containing tetrabutylammonium tetrafluoroborate (TBABF_4_, 0.1 M) as the supporting electrolyte. MeCN was used for the electrochemical experiments with mITO due to the lower solubility of the molecules in MeCN than DMF, which increases their anchoring stability on the electrode. For irreversible oxidations, the half-peak potentials (*E*^(p/2)^) were used to estimate the thermodynamic oxidation potential (*E*(S^+^/S)).^48^ For **DPPdye**, an irreversible oxidation is observed with a potential of +1.22 V *vs.* normal hydrogen electrode (NHE) in DMF solution and of +1.29 V *vs.* NHE for the mITO|**DPPdye** electrode in MeCN (Fig. S8†).

When dissolved in DMF (Fig. S9a†), **DPP-Ru** features a reversible oxidation at +0.60 V *vs.* NHE assigned to the Ru^III^/Ru^II^ couple.^34^ A second oxidation, attributed to the DPP unit, was observed at *E*(S^+^/S) = +1.29 V *vs.* NHE. Upon immobilisation (Fig. S9b†), the Ru^III^/Ru^II^ couple could not be observed, possibly due to the high capacitance of the ITO electrodes or spatial separation from the electrode. The oxidation of the DPP unit was observed at +1.34 V *vs.* NHE, similar to the value recorded in DMF solution.

The electrochemical properties were also confirmed in aqueous sodium acetate (NaOAc, 0.1 M, pH 5.6) solution, in which the oxidation of the chromophore on a mITO|**DPPdye** electrode was observed at +1.29 V *vs.* NHE (Fig. S10a†). A significantly higher current, attributed to catalysis, was obtained for a mITO|**DPP-Ru** electrode (Fig. S10b†).

Thus, the oxidation potential of the DPP unit in both organic and aqueous conditions is more positive than the reported onset of catalysis of [Ru^II^(bda)(pic)_2_] (*E*_cat_ = +1.1 V *vs.* NHE), and should therefore provide sufficient driving force for water oxidation.^34^

Given the energy of the 0–0 transition for **DPPdye** and **DPP-Ru**, (*E*_0–0_ = 2.24 eV, Fig. S6 and S7†), the oxidation potential of the excited chromophore (*E*(S^+^/S*)) in DMF solution can be estimated to be −1.02 and −0.95 V *vs.* NHE, respectively. This allows for sufficient thermodynamic driving force for electron injection into the conduction band of TiO_2_ at a wide range of pH values (*E*_CB_(TiO_2_) = −0.57 V *vs.* NHE at pH 7),^49^ and confirms that the dye meets all of the thermodynamic requirements to be incorporated in a dyad-sensitised photoanode for water oxidation.

### Photoelectrochemistry under sacrificial conditions

PEC experiments were carried out at room temperature in a N_2_-purged one-compartment three electrode electrochemical cell using a platinum counter electrode, a Ag/AgCl/KCl_sat_ reference electrode and a sensitised TiO_2_ film (mTiO_2_, procedure in ESI†) as the working photoelectrode. Linear sweep voltammetry (LSV) experiments were performed under chopped light irradiation and a potential of +0.2 V *vs.* NHE was applied for chronoamperometry experiments. UV-filtered simulated solar light was used for all PEC measurements (100 mW cm^−2^, AM 1.5G, *λ* > 420 nm), avoiding direct excitation of the TiO_2_ semiconductor.

**Fig. 3:**
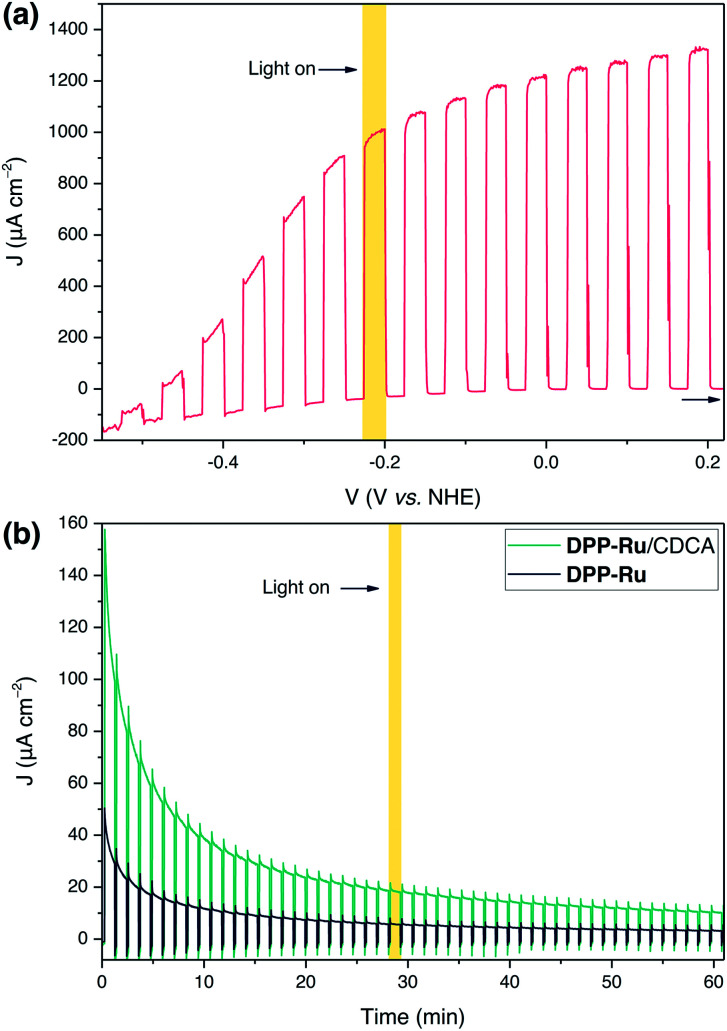
(a) Linear sweep voltammogram of a mTiO_2_|**DPPdye** electrode with chopped illumination (*v* = 5 mV s^−1^). Conditions: aqueous TEOA solution (0.1 M, pH 7), 100 mW cm^−2^, AM 1.5G, *λ* > 420 nm, N_2_ purged, room temperature. (b) Chronoamperometry results at +0.2 V *vs.* NHE under chopped light illumination of TiCl_4_–mTiO_2_|**DPP-Ru** and TiCl_4_–mTiO_2_|**DPP-Ru**/CDCA electrodes incubated in a solution of 0.1 mM **DPP-Ru** in MeOH with varying CDCA concentrations (0 or 20 mM). Conditions: NaOAc buffer (0.1 M, pH 5.6), 100 mW cm^−2^, AM 1.5G, *λ* > 420 nm, N_2_ purged, room temperature.

To evaluate the maximum photocurrent that can be extracted from the dye, without the kinetic limitations of water oxidation catalysis, PEC measurements were performed on a mTiO_2_|**DPPdye** electrode in the presence of triethanolamine (TEOA) as a sacrificial electron donor in aqueous electrolyte solution (0.1 M, pH 7). The photoanodes were prepared by soaking mTiO_2_ electrodes in a solution of **DPPdye** (0.2 mM in DCM) overnight, followed by rinsing and drying in air (see ESI for details†). Photocurrents of up to 1.3 mA cm^−2^ were observed for the mTiO_2_|**DPPdye** electrode (Fig. 3a), which confirms the feasibility of electron injection into the CB of TiO_2_. These currents are much higher than those typically obtained for organic dyes on TiO_2_ electrodes in aqueous conditions with an electron donor, and slightly lower than the ones obtained in aqueous DSSCs, albeit without any electrode or electrolyte optimisation.^17,23,50–54^ During a four hour chronoamperometry experiment (Fig. S11†), a steady decrease of the photocurrent and electrode decolouration was observed, which can be attributed to dye decomposition, and to hydrolysis and desorption of the carboxylate anchoring group at neutral pH.^55,56^

### Photoelectrochemical water oxidation

For water oxidation catalysis, the sacrificial electron donor solution was replaced by an aqueous NaOAc solution (0.1 M, pH 5.6). The mTiO_2_ electrodes were immersed in a solution of **DPP-Ru** (0.1 mM) in MeOH overnight, followed by rinsing and drying in air. During the LSV experiments (Fig. S12a†), photocurrents were observed with an onset of −0.43 V *vs.* NHE, approximately 60 mV more positive than the conduction band of TiO_2_ (*E*_CB_(TiO_2_) = −0.49 V *vs.* NHE at pH 5.6).^49^ At more positive potentials, the photocurrents spike at 200 μA cm^−2^, but quickly decay afterwards. This response can be attributed to an initial fast electron injection from the dye into TiO_2_, followed by charge accumulation and recombination between the electrode and the oxidised dyad.^5,9^ At −0.1 V *vs.* NHE, a net photocurrent of 18 μA cm^−2^ was observed.

To improve the photocurrent response, the TiO_2_ electrodes were treated with a titanium tetrachloride solution (TiCl_4_, TiCl_4_–mTiO_2_, details in ESI†), a straightforward method used to improve the efficiency of DSSCs by increasing the electron diffusion coefficient.^57,58^ PEC experiments were performed as described above, with immobilisation of **DPP-Ru** on TiCl_4_–mTiO_2_ electrodes carried out in different solvents (MeOH, DMF and DCM) to identify the optimised immobilisation conditions (Fig. S13†). In agreement with previous studies using Ru and porphyrin photoabsorbers, in which the immobilisation solvent plays a role in the ordering of the molecules on the surface, and thus on the electron transfer dynamics, higher photocurrents were obtained when using a polar protic solvent.^9,59–61^ The currents observed with TiCl_4_–mTiO_2_|**DPP-Ru** electrodes were significantly higher than for the untreated mTiO_2_|**DPP-Ru** electrodes. Interestingly, the initial photocurrent of the TiCl_4_–mTiO_2_|**DPPdye** electrode was similar to that of the TiCl_4_–mTiO_2_|**DPP-Ru** electrode under identical conditions (Fig. S14a†). However, the UV-visible absorption spectrum of TiCl_4_–mTiO_2_|**DPPdye** after the PEC experiment showed decomposition of the chromophore, whereas the spectrum of TiCl_4_–mTiO_2_|**DPP-Ru** remained largely unchanged (Fig. S14b†). Therefore, the high photocurrents of the TiCl_4_–mTiO_2_|**DPPdye** electrode in the absence of hole scavenger can be attributed to light-driven dye degradation.

The origin of the modest performance of TiCl_4_–mTiO_2_|**DPP-Ru** may be ascribed to aggregation of the dyad on the electrode. The presence of aggregates has been shown to slow down electron injection, and shorten the lifetime of the radical dye cation, leading to a much lower power conversion in DSSCs.^62^ Aggregate formation can be limited by addition of a co-adsorbent, often chenodeoxycholic acid (CDCA), which improves the efficiency of DSSCs despite decreasing the loading of the dye on the electrode.^26,27,31,39^ Furthermore, the use of co-adsorbents can impact the PEC performance by altering the wettability of the electrode or the CB level of TiO_2_.^53,63,64^ While this approach has been successfully implemented to stabilise dyad-sensitised photocathodes for H_2_ evolution,^65^ it has not yet been explored in water oxidising photoanodes.

The loading of the dyad on the surface was optimised in two stages. Firstly, the concentration of CDCA in the immobilisation bath was varied. Despite this leading to a lower **DPP-Ru** loading (Fig. S15a†), higher photocurrents were reached when CDCA was added to the immobilisation bath (Fig. S16†). Similar loading and photocurrents were observed for all concentrations of CDCA studied. The loading of the dyad on the surface and photoanode performance could be further controlled by varying the concentration of **DPP-Ru** in the immobilisation bath while keeping a constant concentration of CDCA (Fig. S17 and S18†). Decreasing the dyad concentration resulted in lower absorbance of the films and lower photocurrents. Furthermore, increasing the concentration resulted in a higher absorbance, but without an accompanied increase in photocurrent response.

Further optimisation of the PEC conditions was attempted by varying the electrolyte solution (Fig. S19†). A similar peak current was obtained in sodium sulfate (0.1 M, pH 7) solution with a faster decrease in photocurrent, which is consistent with hydrolysis of the molecule at neutral pH.

The optimised conditions for PEC experiments were an aqueous NaOAc electrolyte solution (0.1 M, pH 5.6) with a TiCl_4_–mTiO_2_|**DPP-Ru**/CDCA electrode prepared by immobilisation in MeOH solution (0.1 mM **DPP-Ru** and 20 mM CDCA), capable of affording a light harvesting efficiency close to unity up to 530 nm (Fig. S20†). In the chronoamperometry experiment (Fig. 3b), photocurrents of 140 μA cm^−2^ after 10 s illumination are observed in the presence of the co-adsorbent, which represent a 3.5 fold increase compared to PEC experiments in the absence of CDCA.

The decrease in photocurrent could arise from decomposition of the chromophore, implied by the irreversibility of its oxidation, as observed in cyclic voltammetry measurements. Nonetheless, the UV-vis spectra of TiCl_4_–mTiO_2_|**DPP-Ru**/CDCA electrodes after the experiment (Fig. S15b†) show only a slight decrease in the absorption band at 499 nm, suggesting continued integrity of the chromophore on the electrode. The decrease in photocurrent is therefore not attributed to desorption of the molecule or decomposition of the chromophore, but rather to the detachment or decomposition of the WOC. Different deactivation mechanisms have been proposed for [Ru^II^(bda)]-catalysts, which usually occur during the rate-limiting steps when the Ru centre is in the higher oxidation states.^66^ The effect of CDCA addition, which increases the distance between dyad molecules on the electrode surface, is unknown both on the decomposition pathways and the dimerisation pathway, to which the early catalytic onset is attributed.^34^ Further work utilising pump–probe spectroscopic techniques could be used in future studies to gain insight on the role of CDCA in the system.

### Oxygen quantification

To evaluate the faradaic efficiency (FE) of our photoanode for oxygen evolution, collector–generator (CG) cells were fabricated as described previously.^67^ Illumination of a bare TiCl_4_–mTiO_2_ electrode for 10 min (Fig. S21†) leads only to a negligible photocurrent background, due to the 420 nm cut-off filter preventing band gap excitation of the TiO_2_ semiconductor. While a high photocurrent was produced by the TiCl_4_–mTiO_2_|**DPPdye** electrode (Fig. S22†), this only leads to a marginal current increase by the collector, demonstrating that no O_2_ originates from the dye-sensitised electrodes in the absence of the WOC unit.

**Fig. 4:**
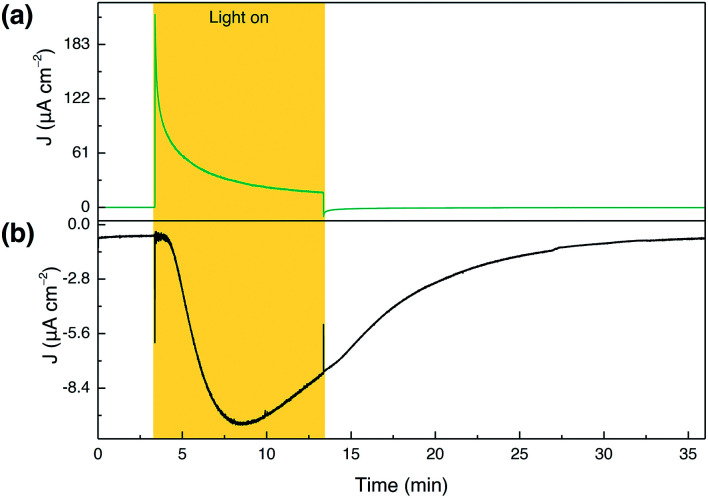
Collector–generator experiment for a TiCl_4_–mTiO_2_|**DPP-Ru**/CDCA electrode prepared by immobilisation in MeOH solution (0.1 mM **DPP-Ru** and 20 mM CDCA). Conditions: NaOAc buffer (0.1 M, pH 5.6) with NaClO_4_ (0.4 M), 100 mW cm^−2^, AM 1.5G, *λ* > 420 nm, N_2_ purged, room temperature. (a) Chronoamperometry photocurrent trace of the generator with an applied potential of +0.2 *vs.* NHE. (b) Chronoamperometry current trace of the collector with an applied potential of −0.6 *vs.* NHE.

The fully assembled TiCl_4_–mTiO_2_|**DPP-Ru**/CDCA electrode displays an initial photocurrent of 140 μA cm^−2^ after 10 s illumination, which decays to 17 μA cm^−2^ over the course of 10 min of PEC operation. In contrast to control experiments, an increase in the O_2_ reduction current by the collector electrode was recorded for the dyad photoanode, corresponding to a FE for O_2_ of 44 ± 3.2% (Fig. 4). Trapped O_2_ in the porous electrode cannot be accounted for and hence lowers collector efficiency (details in the ESI†). In addition, the moderate FE can also be partially attributed to decomposition of the photocatalyst.

### Performance and comparison with state-of-the-art

Inductively coupled plasma optical emission spectrometry (ICP-OES) based on Ru determination, after digestion of fresh TiCl_4_–mTiO_2_|**DPP-Ru**/CDCA electrodes in nitric acid, revealed an initial loading (*Γ*_0_) of 11.7 ± 1.04 nmol cm^−2^ of the dyad. This loading is lower than for other molecules on mesoporous TiO_2_ electrodes, but in line with the large steric footprint of the dyad and the presence of CDCA.^15,45,68,69^ This translates to a turnover number (TON) of 2.3 ± 0.6 for O_2_ evolution for the catalyst (TON_cat_) and 9.2 ± 2 for the dye (TON_dye_). ICP-OES revealed a loading of 7.1 ± 0.7 nmol cm^−2^ of the dyad after the experiment, suggesting catalyst detachment or desorption of the dyad assembly as partly responsible for the decreasing photocurrent.

A molecular dyad made of a zinc porphyrin chromophore and a [Ru^II^(bda)] WOC was previously reported and immobilised on a TiO_2_ electrode.^15^ CV measurements showed that in aqueous conditions the chromophore did not possess sufficient driving force to activate the catalyst. Despite this, during photolysis, O_2_ was measured *via* gas chromatography corresponding to a FE of 33% and a TON_cat_ of 1.3, but the role of direct excitation of TiO_2_ was not ruled out under the employed experimental conditions. A Ru dye-[Ru^II^(bda)] catalyst dyad was able to evolve O_2_ with a FE of 30% on TiO_2_ and 74% on SnO_2_/TiO_2_ electrodes.^11^ The FE values for O_2_ evolution reported here compare favourably to the precious-metal chromophore dyad, and show for the first time catalytic turnover of both catalyst and dye using a dyad with a metal-free chromophore. The infancy of organic chromophores compared to Ru dyes for PEC in aqueous conditions is reflected in the superior stability of the Ru-dye based water oxidation dyad, but future organic chromophore development and optimisation opens the door to the replacement of precious metal with earth-abundant chromophores.

Despite higher dye loadings, photocurrents obtained for co-immobilised systems with organic push–pull dyes on SnO_2_/TiO_2_ electrodes are typically low and FEs for O_2_ evolution are in the range of 10%.^17,23^ Embedding the chromophore in a thin metal oxide layer by atomic layer deposition (∼1 nm, TiO_2_ or Al_2_O_3_) and further immobilisation of the catalyst has been studied as an alternative way of limiting oxidative decomposition of the chromophore.^18,19,22^ Improved efficiencies, between 11% and 49%, were thus reached. However, comparison with these systems is limited since TON values have not been reported. A TON_cat_ of 3.0 and a TON_dye_ of 2.4 were reported for a borondipyrromethene chromophore co-immobilised with a functionalised [Ru^II^(bda)] catalyst on a TiO_2_ electrode, with a FE for O_2_ of 77%.^21^ When a subporphyrin dye was employed with an analogous catalyst, a TON_cat_ of 27 and a TON_dye_ of 14 were obtained with a FE of 64%.^20^ Thus, the results obtained highlight the benefit of dyadic systems compared to a co-immobilised approach in making an efficient use of dye molecules relative to the catalyst.

## Conclusions

We present an organic dye-ruthenium catalyst dyad consisting of a DPP dye and a [Ru^II^(bda)]-type complex. The chromophore displayed strong light absorption in the visible part of the electromagnetic spectrum, and suitable thermodynamics for electron injection into the conduction band of TiO_2_ and hole transfer to the Ru WOC. The dyad was then integrated in a TiO_2_-based photoanode for light-driven water oxidation. Incorporation of CDCA as a co-adsorbent was shown to significantly increase the photocurrents from 40 to 140 μA cm^−2^ in aqueous sodium acetate solution (0.1 M, pH 5.6), despite a lower dyad loading. The FE for O_2_ evolution was found to be 44% and corresponds to a TON_cat_ of 2.3 and a TON_dye_ of 9.2. UV-vis absorption measurements indicated that the decrease in current during photolysis was mainly associated to catalyst detachment or decomposition rather than dyad desorption or chromophore decomposition. This study shows that a metal-free dye with sufficient oxidising power can be covalently linked to a molecular catalyst for catalytic O_2_ evolution on a dyad-sensitised photoanode. Key techniques for accommodating chromophores with strong intermolecular π–π stacking interactions have been highlighted, and the significant benefits of CDCA co-adsorption on molecular dye-sensitised photoanodes for water oxidation has been demonstrated. Future experiments with time-resolved spectroscopy can be used to gain insight on the role of CDCA in enhancing the PEC performance, and serve as a blueprint for subsequent molecular design.

## Conflicts of interest

There are no conflicts to declare.

## Supplementary Material

SC-011-D0SC04509H-s001

## References

[cit1] Li F., Yang H., Li W., Sun L. (2018). Joule.

[cit2] Zhang B., Sun L. (2019). Chem. Soc. Rev..

[cit3] Brennaman M. K., Dillon R. J., Alibabaei L., Gish M. K., Dares C. J., Ashford D. L., House R. L., Meyer G. J., Papanikolas J. M., Meyer T. J. (2016). J. Am. Chem. Soc..

[cit4] Swierk J. R., McCool N. S., Mallouk T. E. (2015). J. Phys. Chem. C.

[cit5] Zhao Y., Swierk J. R., Megiatto, Jr. J. D., Sherman B., Youngblood W. J., Qin D., Lentz D. M., Moore A. L., Moore T. A., Gust D., Mallouk T. E. (2012). Proc. Natl. Acad. Sci. U. S. A..

[cit6] Song W., Ito A., Binstead R. A., Hanson K., Luo H., Brennaman M. K., Concepcion J. J., Meyer T. J. (2013). J. Am. Chem. Soc..

[cit7] Zhang B., Sun L. (2019). J. Am. Chem. Soc..

[cit8] Duan L., Fischer A., Xu Y., Sun L. (2009). J. Am. Chem. Soc..

[cit9] Swierk J. R., McCool N. S., Saunders T. P., Barber G. D., Mallouk T. E. (2014). J. Am. Chem. Soc..

[cit10] Ashford D. L., Song W., Concepcion J. J., Glasson C. R. K., Brennaman M. K., Norris M. R., Fang Z., Templeton J. L., Meyer T. J. (2012). J. Am. Chem. Soc..

[cit11] Sherman B. D., Xie Y., Sheridan M. V., Wang D., Shaffer D. W., Meyer T. J., Concepcion J. J. (2017). ACS Energy Lett..

[cit12] Ashford D. L., Sherman B. D., Binstead R. A., Templeton J. L., Meyer T. J. (2015). Angew. Chem., Int. Ed..

[cit13] Sherman B. D., Sheridan M. V., Wee K.-R., Marquard S. L., Wang D., Alibabaei L., Ashford D. L., Meyer T. J. (2016). J. Am. Chem. Soc..

[cit14] Sherman B. D., Ashford D. L., Lapides A. M., Sheridan M. V., Wee K.-R., Meyer T. J. (2015). J. Phys. Chem. Lett..

[cit15] Yamamoto M., Wang L., Li F., Fukushima T., Tanaka K., Sun L., Imahori H. (2016). Chem. Sci..

[cit16] Decavoli C., Boldrini C. L., Manfredi N., Abbotto A. (2020). Eur. J. Inorg. Chem..

[cit17] Eom Y. K., Nhon L., Leem G., Sherman B. D., Wang D., Troian-Gautier L., Kim S., Kim J., Meyer T. J., Reynolds J. R., Schanze K. S. (2018). ACS Energy Lett..

[cit18] Wang D., Eberhart M. S., Sheridan M. V., Hu K., Sherman B. D., Nayak A., Wang Y., Marquard S. L., Dares C. J., Meyer T. J. (2018). Proc. Natl. Acad. Sci. U. S. A..

[cit19] Alibabaei L., Dillon R. J., Reilly C. E., Brennaman M. K., Wee K.-R., Marquard S. L., Papanikolas J. M., Meyer T. J. (2017). ACS Appl. Mater. Interfaces.

[cit20] Yamamoto M., Nishizawa Y., Chábera P., Li F., Pascher T., Sundström V., Sun L., Imahori H. (2016). Chem. Commun..

[cit21] Suryani O., Higashino Y., Mulyana J. Y., Kaneko M., Hoshi T., Shigaki K., Kubo Y. (2017). Chem. Commun..

[cit22] Na Y., Miao S., Zhou L., Wei P., Cao Y. (2018). Sustainable Energy Fuels.

[cit23] Wee K.-R., Sherman B. D., Brennaman M. K., Sheridan M. V., Nayak A., Alibabaei L., Meyer T. J. (2016). J. Mater. Chem. A.

[cit24] Grzybowski M., Gryko D. T. (2015). Adv. Opt. Mater..

[cit25] Chandran D., Lee K.-S. (2013). Macromol. Res..

[cit26] Ganesan P., Yella A., Holcombe T. W., Gao P., Rajalingam R., Al-Muhtaseb S. A., Grätzel M., Nazeeruddin M. K. (2015). ACS Sustainable Chem. Eng..

[cit27] Favereau L., Warnan J., Pellegrin Y., Blart E., Boujtita M., Jacquemin D., Odobel F. (2013). Chem. Commun..

[cit28] Farré Y., Zhang L., Pellegrin Y., Planchat A., Blart E., Boujtita M., Hammarström L., Jacquemin D., Odobel F. (2016). J. Phys. Chem. C.

[cit29] Qu S., Wu W., Hua J., Kong C., Long Y., Tian H. (2010). J. Phys. Chem. C.

[cit30] Wiberg J., Marinado T., Hagberg D. P., Sun L., Hagfeldt A., Albinsson B. (2009). J. Phys. Chem. C.

[cit31] Holcombe T. W., Yum J.-H., Kim Y., Rakstys K., Grätzel M. (2013). J. Mater. Chem. A.

[cit32] Warnan J., Willkomm J., Ng J. N., Godin R., Prantl S., Durrant J. R., Reisner E. (2017). Chem. Sci..

[cit33] Creissen C. E., Warnan J., Reisner E. (2018). Chem. Sci..

[cit34] Duan L., Bozoglian F., Mandal S., Stewart B., Privalov T., Llobet A., Sun L. (2012). Nat. Chem..

[cit35] Lebedev D., Pineda-Galvan Y., Tokimaru Y., Fedorov A., Kaeffer N., Copéret C., Pushkar Y. (2018). J. Am. Chem. Soc..

[cit36] Concepcion J. J., Zhong D. K., Szalda D. J., Muckerman J. T., Fujita E. (2015). Chem. Commun..

[cit37] Tan C.-J., Yang C.-S., Sheng Y.-C., Amini H. W., Tsai H.-H. G. (2016). J. Phys. Chem. C.

[cit38] Qu S., Tian H. (2012). Chem. Commun..

[cit39] Warnan J., Favereau L., Pellegrin Y., Blart E., Jacquemin D., Odobel F. (2011). J. Photochem. Photobiol., A.

[cit40] Gao Y., Ding X., Liu J., Wang L., Lu Z., Li L., Sun L. (2013). J. Am. Chem. Soc..

[cit41] Zhang B., Li F., Zhang R., Ma C., Chen L., Sun L. (2016). Chem. Commun..

[cit42] Dhar J., Venkatramaiah N., Anitha A., Patil S. (2014). J. Mater. Chem. C.

[cit43] Escudero D. (2016). Acc. Chem. Res..

[cit44] Rosser T. E., Reisner E. (2017). ACS Catal..

[cit45] Leung J. J., Warnan J., Ly K. H., Heidary N., Nam D. H., Kuehnel M. F., Reisner E. (2019). Nat. Catal..

[cit46] Muresan N. M., Willkomm J., Mersch D., Vaynzof Y., Reisner E. (2012). Angew. Chem., Int. Ed..

[cit47] Hoertz P. G., Chen Z., Kent C. A., Meyer T. J. (2010). Inorg. Chem..

[cit48] Espinoza E. M., Clark J. A., Soliman J., Derr J. B., Morales M., Vullev V. I. (2019). J. Electrochem. Soc..

[cit49] Kavan L., Tétreault N., Moehl T., Grätzel M. (2014). J. Phys. Chem. C.

[cit50] Warnan J., Willkomm J., Farré Y., Pellegrin Y., Boujtita M., Odobel F., Reisner E. (2019). Chem. Sci..

[cit51] Bella F., Gerbaldi C., Barolo C., Grätzel M. (2015). Chem. Soc. Rev..

[cit52] Leandri V., Ellis H., Gabrielsson E., Sun L., Boschloo G., Hagfeldt A. (2014). Phys. Chem. Chem. Phys..

[cit53] Law C., Moudam O., Villarroya-Lidon S., O'Regan B. (2012). J. Mater. Chem..

[cit54] Bella F., Porcarelli L., Mantione D., Gerbaldi C., Barolo C., Grätzel M., Mecerreyes D. (2020). Chem. Sci..

[cit55] Bae E., Choi W., Park J., Shin H. S., Bin Kim S., Lee J. S. (2004). J. Phys. Chem. B.

[cit56] Materna K. L., Crabtree R. H., Brudvig G. W. (2017). Chem. Soc. Rev..

[cit57] O'Regan B. C., Durrant J. R., Sommeling P. M., Bakker N. J. (2007). J. Phys. Chem. C.

[cit58] Lee S.-W., Ahn K.-S., Zhu K., Neale N. R., Frank A. J. (2012). J. Phys. Chem. C.

[cit59] Imahori H., Hayashi S., Hayashi H., Oguro A., Eu S., Umeyama T., Matano Y. (2009). J. Phys. Chem. C.

[cit60] Imahori H., Kang S., Hayashi H., Haruta M., Kurata H., Isoda S., Canton S. E., Infahsaeng Y., Kathiravan A., Pascher T., Chábera P., Yartsev A. P., Sundström V. (2011). J. Phys. Chem. A.

[cit61] Ye S., Kathiravan A., Hayashi H., Tong Y., Infahsaeng Y., Chabera P., Pascher T., Yartsev A. P., Isoda S., Imahori H., Sundström V. (2013). J. Phys. Chem. C.

[cit62] de Miguel G., Marchena M., Ziółek M., Pandey S. S., Hayase S., Douhal A. (2012). J. Phys. Chem. C.

[cit63] Kay A., Grätzel M. (1993). J. Phys. Chem..

[cit64] Neale N. R., Kopidakis N., van de Lagemaat J., Grätzel M., Frank A. J. (2005). J. Phys. Chem. B.

[cit65] Kaeffer N., Massin J., Lebrun C., Renault O., Chavarot-Kerlidou M., Artero V. (2016). J. Am. Chem. Soc..

[cit66] Duan L., Araujo C. M., Ahlquist M. S. G., Sun L. (2012). Proc. Natl. Acad. Sci. U. S. A..

[cit67] Sherman B. D., Sheridan M. V., Dares C. J., Meyer T. J. (2016). Anal. Chem..

[cit68] Leung J. J., Warnan J., Nam D. H., Zhang J. Z., Willkomm J., Reisner E. (2017). Chem. Sci..

[cit69] Schreier M., Luo J., Gao P., Moehl T., Mayer M. T., Grätzel M. (2016). J. Am. Chem. Soc..

