# Supplementary material for: A diketopyrrolopyrrole dye-based dyad on a porous TiO2 photoanode for solar-driven water oxidation
Source: Chem Sci. 2020 Sep 25;11(47):12769–76. doi: 10.1039/d0sc04509h (PMC8163027; doi:10.1039/d0sc04509h)
Supplement: SC-011-D0SC04509H-s001 [file SC-011-D0SC04509H-s001.pdf]

Electronic Supplementary Material (ESI) for Chemical Science.
This journal is © The Royal Society of Chemistry 2020

Electronic Supplementary Information

A diketopyrrolopyrrole dye-based dyad on a porous TiO_2 photoanode for solar-driven water oxidation

Daniel Antón-García,[a] Julien Warnan,[a] and Erwin Reisner*,[a]

[a] Department of Chemistry, University of Cambridge, Lensfield Road, Cambridge CB2 1EW, United Kingdom

*Correspondence to: reisner@ch.cam.ac.uk

Contents

Experimental section S2
Supporting figures S9
Supporting references S22

Experimental section

Materials and methods

Chemicals for synthesis and analytical measurements were of the highest available purity. Acetonitrile (MeCN), dichloromethane (DCM), methanol (MeOH), piperidine, tetrahydrofuran (THF) and triethylamine (TEA) were distilled on calcium hydride (TEA, MeOH & DCM), potassium hydroxide (piperidine) or sodium (THF) before use. Dry 1,4-dioxane (99.8%) and *N*,*N*-dimethylformamide (DMF, 99.8%) were purchased from Sigma Aldrich. Column chromatography was carried out with silica gel 60 (0.040-0.063 mm mesh) from Material Harvest. Fluorine-doped tin oxide (FTO)-coated glass sheets were purchased from Sigma Aldrich (SnO_2/F, 7Ωsq^{-1} sheet resistance, 300 × 300 × 2 mm). ITO nanopowder was obtained from Sigma Aldrich (diameter < 50 nm; BET = 27 m^2 g^{-1} ; 90% In_2O_3, 10% SnO_2). The TiO_2 paste (15-20 nm, Ti-Nanoxide T/SP, 100% anatase) was purchased from Solaronix. All aqueous solutions were prepared with ultrapure water (DI water; Milli-Q®, 18.2 MΩ cm).

Physical methods

^{1}H, ^{13}C and ^{11}B NMR spectra were recorded on a Bruker DPX-400 MHz or a Bruker 500 MHz DCH cryoprobe spectrometer at room temperature. Chemical shifts are given in ppm and coupling constants in Hz. Chemical shifts for ^{1}H NMR spectra are referenced relative to residual protons in the deuterated solvent ($CHCl_3$: δH = 7.26 ppm; CD_2Cl_2: δH = 5.32 ppm; THF: δH = 1.72 ppm). High resolution-mass spectra were recorded using a ThermoScientific Orbitrap Classic mass spectrometer. UV-vis spectra were collected in triplicate using a Agilent Cary 60 UV-vis Spectrophotometer. For solution spectra, a quartz cuvette (Hellma, 1 cm path length) was used. Elemental analysis was carried out by the Microanalysis Service of the Department of Chemistry, University of Cambridge, using an Exeter Analytical CE-440 Elemental Analyzer. Inductively coupled plasma optical emission spectrometry (ICP-OES) measurements were also conducted by the Microanalysis Service, on a Thermo Scientific iCAP 7400 ICPOES DUO spectrometer. Infrared spectra were recorded on a Thermo Scientific Nicolet iS50 FT-IR spectrometer in ATR (attenuated total reflection) mode. A IA9000 series Digital Melting Point Apparatus, provided by the Part IB teaching labs from the Department of Chemistry, was used for melting point determination. Fluorescence spectroscopy was recorded on an Edinburgh Instruments FS5 Spectrofluorometer using a Suprasil Quartz (QS) cuvette with 1 cm path length at room temperature. Fluorescence quantum yield measurements were performed using an integrating sphere and samples with an optical density in the range of 0.06-0.12.

Electrode preparation

Preparation of ITO electrodes (mITO)

FTO-coated glass was cleaned by immersing it in a solution of H_2O/37% NH_3/30% H_2O_2 (50:17:33 v/v) at 80 °C for 15 min. The electrodes were rinsed with water and successive sonication in ethanol and acetone for 15 minutes each, followed by drying at 70 °C in air before further use. A dispersion of 20% weight ITO nanoparticles was prepared in 5 M acetic acid in ethanol solution. After sonication for 30 minutes in an ice-cooled sonication bath, the ITO thin films were spin-

coated onto the cleaned FTO-glass substrates. The electrodes were finally sintered at 450 °C for 30 minutes. The electrodes had a geometrical area of approximately 1 x 2 cm^2.[1]

Preparation of TiO_2 films (mTiO_2)

FTO-coated glass was cleaned by immersing it in a solution of H_2O/ 37% NH_3/ 30% H_2O_2 (50:17:33 v/v) at 80 °C for 15 minutes. The electrodes were rinsed with water and successive sonication in ethanol and acetone for 15 minutes each, followed by drying at 70 °C in air before further use. The mesoporous TiO_2 scaffold was deposited by slot-coating commercial Ti-Nanoxide pastes (15-20 nm particles, 100 % anatase, Solaronix) over a defined area (approximately 0.6 × 0.6 cm). The electrodes were then sintered in a Carbolite furnace, under atmospheric conditions using the following heating ramp. Immediately after slot-coating, the electrodes were transferred into a furnace pre-heated at 135 °C. The temperature was increased to 325 °C and held at this temperature for 5 min, after which the temperature was further augmented to 375 °C and maintained for 5 min at this temperature. The temperature was finally allowed to reach 475 °C for 40 minutes. The electrodes were removed from the furnace after a slow cooling period. The electrodes had an approximate thickness of 6 µm.[1]

Preparation of TiO_2 films treated with $TiCl_4$ ($TiCl_4$-mTiO_2)

The $TiCl_4$ treatment of the FTO was conducted by first preparing a 2 M $TiCl_4$ solution in H_2O. After dilution of the mother $TiCl_4$ solution (2 mL in 98 mL of distilled water), the FTO substrates were immersed into the solution and heated to 70 °C for 30 min. The electrodes were then rinsed in water, ethanol and allowed to dry in air. The TiO_2 film was deposited as described above, sintered and a second identical $TiCl_4$ treatment was given. A final firing at 475 °C for a further 40 min was performed and the electodes were removed from the furnace after a slow cooling period.

Immobilisation of molecules on electrodes

The electrode was immersed overnight at room temperature in the dark in a bath of **DPP_{dye}** (0.2 mM) in DCM or **DPP-Ru** and CDCA (varying concentrations) in MeOH, DMF or DCM. The electrodes were then rinsed in the immobilisation solvent, followed by DCM and then dried in air.

Electrochemistry experiments

Electrochemistry was carried out using an Ivium CompactStat potentiostat. Measurements were performed at room temperature under N_2 in a three electrode configuration. All electrolyte solutions were purged with N_2 for 15 min prior to the measurements to remove O_2. A glassy carbon disk (3 mm diameter) or sensitised mITO working electrode, and a Pt mesh counter electrode were employed. Measurements in DMF/$TBABF_4$ or MeCN/$TBABF_4$ electrolyte solution ($TBABF_4$ = tetrabutylammonium tetrafluoroborate, 0.1 M) were performed using a Ag wire reference electrode. Ferrocene was added as an internal standard and the redox potentials were referenced against the Fc^+/Fc redox couple. The conversion factor used from the Fc^+/Fc couple to NHE was +0.720 V in DMF and +0.690 V in MeCN.[2] For measurements in aqueous conditions, a Pt-counter electrode and a Ag/AgCl/KCl_{sat} reference were used. The potentials have been quoted *vs.* NHE using the conversion $E_{NHE}=E_{Ag/AgCl}$ +0.199. Redox potentials are given as $E_{1/2}$ and were

determined from the cathodic and anodic peak potentials (for reversible redox couples) and as the half-peak potentials ($E^{(p/2)}$)) for irreversible redox waves.

Photoelectrochemistry experiments

Photoelectrochemical measurements were carried out using an Ivium CompactStat potentiostat in a one-compartment three necked custom-made cell equipped with a flat borosilicate glass window. A three-electrode setup was used with a Pt-counter electrode, a Ag/AgCl/KCl_{sat} reference, and the working electrode consisted of the sensitised $mTiO_2$ or $TiCl_4$-$mTiO_2$ electrodes. All potentials have been quoted *vs.* NHE using the conversion $E_{NHE}=E_{Ag}/_{AgCl}$ +0.199. The cell was purged with N_2 for 15 min prior to the measurements. Back illumination was used for all experiments using a calibrated Newport Oriel solar light simulator (150 W, 100 mW cm^{-2} across the solar spectrum, AM 1.5G) fitted with a UQG Optics UV Filter (λ > 420 nm) and IR water filter. LSVs were conducted at a scan rate of 5 mV s^{-1} with chopped light alternating between dark and light every 5 seconds, followed by the chronoamperometry measurement.

Oxygen quantification using collector-generator cells

The collector-generator electrode set-up for oxygen detection was adapted from the literature.[3] The collector electrode (FTO_{COL}) consisted of an unmodified FTO-glass (4 x 1.5 cm). The dye-sensitied generator electrode (FTO_{GEN}) consisted of approximately 0.75 x 1.1 cm $TiCl_4$-$mTiO_2$ films which were prepared as described previously. 1 mm thick non-conductive microscope slides were glued as a spacer to the bottom right and left corners of the conductive face of the FTO_{COL} using Araldite 5-Minute Rapid epoxy and left overnight. Using the same epoxy, the sensitised FTO_{GEN} was bonded to the collector with both conductive sides facing each other.

An Alvatek Ivium CompactStat equipped with bipotentiostat capabilities was used for all measurements in a two necked custom-made cell equipped with a flat borosilicate glass window. A Ag/AgCl/KCl_{sat} reference was placed near the working electrodes and a platinum counter electrode was placed separated from the working solution by a nafion membrane. A solution of sodium acetate (0.1 M, pH 5.6) buffer was used with sodium perchlorate ($NaClO_4$, 0.4 M) as a supporting electrolyte to avoid capacitance between the working electrodes.

The cell was purged with N_2 for 15 min, after which a potential of −0.60 V *vs.* NHE was applied to the FTO_{COL} for a minimum of 20 minutes and until any residual oxygen had been reduced. During the experiment, a potential of +0.2 V and −0.60 V *vs.* NHE were applied to the FTO_{GEN} and FTO_{COL} respectively. Back illumination on the FTO_{GEN} side was used for all experiments for a period of 10 minutes using a calibrated Newport Oriel solar light simulator (150 W, 100 mW cm^{-2}, AM 1.5G) fitted with a UQG Optics UV Filter (λ > 420 nm) and IR water filter. After the illumination period, the potentials were held until all oxygen had been reduced by the FTO_{COL}.

The collector efficiency ($\eta_{col\ eff}$) was calculated by applying a potential of +1.67 *vs.* NHE to the FTO electode (FTO_{GEN}) for 500 seconds while a reducing potential of −0.60 *vs.* NHE was applied to the FTO_{COL} for a further 20 minutes until the current returned to baseline. The collector efficiency was determined individually for each electrode and ranged between 68% and 76%.

Experiments for the fully assembled $TiCl_4$-$mTiO_2$|**DPP-Ru**/CDCA system were conducted in triplicate.

Quantification of immobilised molecules

The molecular surface coverage (Γ_0) of **DPP-Ru** was determined in triplicate by inductively coupled plasma - optical emission spectrometry (ICP-OES) based on Ru determination, following digestion of electrodes in aqueous HNO_3 (70%) during the course of three days, and subsequent dilution to achieve a final concentration of 7% HNO_3.

Faradaic efficiency and TON determination

The Faradaic efficiency for oxygen evolution (η_{O_2}) was determined directly using the charge (Q) passed at each electrode and according to the following equation:

$$\eta_{O_2} = \frac{\left(\frac{Q_{COL}}{Q_{GEN}}\right)}{\eta_{col\,eff}} \qquad (1)$$

The number of moles of O_2 (nO_2) evolved was calculated using the following equation:

$$nO_2 = \frac{Q_{COL} \times \eta_{O_2}}{4F} \qquad (2)$$

Where F is Faraday's constant (F = 96485.33 C mol^{-1}) and 4 indicates a 4-electron reaction.

The turnover number (TON) of the catalyst (TON_{cat}) and of the dye (TON_{dye}) were calculated as follows:

$$TON_{cat} = \frac{nO_2}{\Gamma_0 A} \qquad (3)$$

$$TON_{dye} = 4 \times TON_{cat} \qquad (4)$$

Where A is the geometric area of the $TiCl_4$-$mTiO_2$ film.

Mean and standard deviation

$$x_u = \sum_i \frac{x_i}{n} \qquad \sigma = \sqrt{\sum_i \frac{(x_i - x_u)^2}{(n-1)}} \qquad (5)$$

Synthesis and characterisation of molecules

DPP$_I$ was prepared as previously described.[4] **Ru$_{WOC}$** ([Ru^{II}(bda)(dmso)(pic)]) was synthesised as previously reported in the literature.[5] [Ru^{II}(bda)(pic)$_2$] was isolated as a side product from the synthesis of **Ru$_{WOC}$** and the spectra matched what was previously reported.[6]

Synthesis of S1

4-picoline (0.7 mL, 7.20 mmol) was added to dry THF (60 mL) and the mixture was bubbled with nitrogen for 10 min and cooled in an ice bath. Lithium diisopropylamide (LDA) (3.96 mL, 7.9 mmol, 2 M solution in hexane/THF) was added via a syringe dropwise and the ice bath was replaced by acetone/dry ice. 4-bromobenzyl bromide (1.50 g, 6.00 mmol) was dissolved in 30 mL dry THF and degassed for 10 min in a Schlenk flask. The mixture was transferred over the course of 10 min. The reaction mixture was kept at −78 °C for 1.5 h before removing the acetone/dry ice bath and letting it warm up to room temperature overnight. The reaction was quenched with water, filtered and the solvent was removed under reduced pressure. The yellow solid was dissolved in chloroform, treated with activated charcoal, filtered and the solvent was removed under reduced pressure to afford **S1** as an off-white solid. (1.08 g, 68%). **Mp:** 87 °C. **^{1}H NMR (400 MHz, chloroform-*d*) δ:** 8.48 (dd, *J* = 4.5, 1.6 Hz, 2H), 7.38 (td, *J* = 9.1, 2.0 Hz, 2H), 7.05 (td, *J* = 4.4, 4.0, 1.6 Hz, 2H), 6.99 (td, *J* = 8.8, 1.8 Hz, 2H), 2.88 (s, 4H). **^{13}C NMR (101 MHz, chloroform-d) δ:** 150.2, 149.8, 139.6, 131.6, 130.3, 124.0, 120.2, 36.9, 36.0. **FT-IR (ATR) σ/cm^{-1}:** 3073, 3027, 2989, 2946, 2927, 2858, 2848, 1596, 1557, 1487, 1450, 1414, 1402, 1221, 1010, 992, 864, 813, 804, 742, 522, 485. **HRMS (ESI/FTMS) m/z:** [M + H]$^+$ Calcd for $C_{13}H_{13}NBr$ 262.0231; Found 262.0234. **Anal.** calcd. for $C_{13}H_{12}BrN$: C, 59.56; H, 4.61; N, 5.34 found: C, 59.62; H, 4.64; N, 5.43.

Synthesis of 1

S1 (518 mg, 1.98 mmol), bis(pinacolato)diboron (1.15 g, 4.54 mmol), potassium acetate (446 mg, 4.54 mmol) and [1,1′-Bis(diphenylphosphino)ferrocene] dichloropalladium(II) (86.70 mg, 1.19 x 10^{-4} mol) were added to 20 mL of dry 1,4-dioxane under a N_2 atmosphere. The mixture was heated to 80 °C overnight, cooled down and after addition of water it was extracted with DCM three times. The organic extracts were combined, washed with water two times and dried with magnesium sulfate. The solvent was removed under reduced pressure and the crude product was purified by flash chromatography (5:5 DCM/ ethyl acetate) to afford **1** as an off-white solid. (412 mg, 73%). **Mp:** 135°C. **^{11}B NMR (128 MHz, chloroform-d) δ:** 30.53. **^{1}H NMR (400 MHz, Chloroform-d) δ:** 8.51 – 8.44 (m, 2H), 7.77-7.69 (m, 2H), 7.20-7.12 (m, 2H), 7.10-7.03 (m, 2H), 3.00-2.86 (m, 4H), 1.34 (s, 12H). **^{13}C NMR (101 MHz, chloroform-*d*) δ:** 150.4, 149.9, 144.1, 135.1, 128.0, 124.1, 83.9, 37.0, 36.9, 25.0. **FT-IR (ATR) σ/cm^{-1}:** 3000, 2978, 2923, 1612, 1600, 1560, 1407, 1398, 1356, 1326, 1275, 1169, 1143, 1091, 1022, 991, 964, 860, 824, 801, 672, 656, 630. **HRMS (ESI/FTMS) m/z:** [M + H]$^+$ Calcd for $C_{19}H_{25}NO_2B$ 310.1978; Found 310.1972. **Anal.** calcd. for $C_{19}H_{24}BNO_2$: C, 73.80; H, 7.82; N, 4.53 found: C, 73.55; H, 7.80; N, 4.51.

Synthesis of DPP$_{II}$

DPP$_I$ (101 mg, 1.43 x 10^{-4} mol), sodium carbonate (152 mg, 1.43 mmol), **2** (53.4 mg, 1.73 x 10^{-4} mol), and tetrakis(triphenylphosphine)palladium(0) (8.32 mg, 7.20 x 10^{-6} mol) were solubilised in

4:1 THF/ water (32 mL) in a N_2 atmosphere. The mixture was then heated for 16 h at 80 °C before cooling down and removing the solvent under reduced pressure. Water and DCM were added, and the product was extracted until the aqueous layer remained colourless. The organic fractions were then washed with water, dried with magnesium sulfate and the solvent was removed under reduced pressure. The crude was then purified with flash chromatography (DCM – DCM/Ethyl acetate 75:25) to afford **DPP_{II}** as a red solid (110 mg, 95%). **Mp:** 114°C. **^{1}H NMR (400 MHz, chloroform-d) δ:** 9.92 (s, 1H), 8.54-8.48 (m, 2H), 7.92-7.85 (m, 4H), 7.83-7.79 (m, 2H), 7.77 (d, *J* = 3.9 Hz, 1H), 7.74-7.70 (m, 2H), 7.58-7.54 (m, 2H), 7.15-7.08 (m, 2H), 3.86-3.71 (m, 4H), 3.06-2.94 (m, 4H), 1.59-1.48 (m, 2H), 1.25-1.01 (m, 17H), 0.81-0.71 (m, 12H). **^{13}C NMR (101 MHz, chloroform-*d*) δ:** 182.9, 163.0, 162.8, 152.8, 150.5, 149.9, 147.2, 143.5, 143.4, 140.9, 138.0, 137.4, 129.6, 129.5, 129.3, 129.2, 127.3, 127.3, 127.3, 126.8, 125.1, 124.1, 110.0, 45.3, 38.8, 38.7, 37.1, 36.4, 30.5, 28.4, 28.4, 23.9, 23.0, 23.0, 14.1, 10.6, 10.6. **FT-IR (ATR) σ/cm^{-1}:** 2956, 2926, 2869, 2857, 1663, 1598, 1528, 1496, 1447, 1414, 1389, 1370, 1356, 1220, 1089, 818, 807. **HRMS (ESI/FTMS) m/z:** $[M + H]^+$ Calcd for $C_{52}H_{58}O_3N_3{}^{32}S$ 804.4193; Found 804.4197.

Synthesis of DPP_{dye}

DPP_{II} (93.1 mg, 1.16 x 10^{-4} mol), piperidine (473 mg, 5.56 mmol) and cyanoacetic acid (374 mg, 4.40 mmol) were dissolved in dry THF under a N_2 atmosphere. The mixture was refluxed for 10 h and cooled to room temperature. Dilute HCl was added and the mixture was extracted three times with DCM. The organic fractions were washed with water, dried with magnesium sulfate and the solvent was removed under reduced pressure. The crude was purified by flash chromatography (DCM – DCM/MeOH 94:6 – DCM/MeOH/TEA 92:8: 4 drops/ 10mL). The product was then washed with dilute HCl and the solvent was removed under reduced pressure to afford **DPP_{dye}** (85 mg, 84%) as a red solid. **Mp:** 163°C. **^{1}H NMR (400 MHz, THF-*d*$_8$) δ:** 8.44-8.40 (m, 2H), 8.39 (s, 1H), 8.08-7.94 (m, 7H), 7.92 (d, *J* = 4.0 Hz, 1H), 7.82 (d, *J* = 8.3 Hz, 3H), 7.74 (d, *J* = 4.0 Hz, 1H), 7.68 (s, 1H), 7.31 (d, *J* = 8.0 Hz, 3H), 7.16-7.13 (m, 2H), 3.94-3.83 (m, 4H), 3.05-2.91 (m, 4H), 1.55-1.48 (s, 2H), 1.28 – 1.11 (m, 17H), 0.82-0.73 (m, 12H). **^{13}C NMR (126 MHz, THF-*d*$_8$) δ:** 162.9, 162.0, 161.9, 151.8, 149.9, 149.6, 147.9, 146.3, 145.5, 143.1, 141.2, 138.9, 137.6, 136.2, 134.9, 115.5, 110.38, 109.81, 99.8, 44.4, 44.4, 38.7, 38.7, 38.6, 38.6, 36.6, 36.1, 30.3, 28.2, 28.1, 23.6, 23.6, 22.7, 22.7, 13.3, 9.74. **UV-vis (DMF) λ_{max}/nm (ε × 10^{-3} /M^{-1} cm^{-1}):** 327(18.40), 374(sh, 25.08), 390(27.18), 499(27.95). **FT-IR (ATR) σ/cm^{-1}:** 2955, 2925, 2869, 2856, 2213, 2161, 1668, 1637, 1585, 1496, 1457, 1438, 1388, 1380, 1354, 1262, 1213, 1089, 1004, 818, 743. **HRMS (ESI/FTMS) m/z:** $[M + H]^+$ Calcd for $C_{55}H_{59}O_4N_3{}^{32}S$ 871.4252; Found 871.4244. **Anal.** calcd. for $C_{55}H_{58}N_4O_4S{\cdot}H_2O$: C, 74.29; H, 6.80; N, 6.30 found: C, 74.68; H, 6.52; N, 6.29.

Synthesis of DPP-Ru

DPP_{dye} (38.1 mg, 4.37 x 10^{-5} mol), $[Ru^{II}$(bda)(dmso)(picoline)] (31.5 mg, 6.12 x 10^{-5} mol) were mixed in 10 mL of dry MeOH with a couple of drops of triethylamine in the dark under a N_2 atmosphere, and the mixture was stirred under reflux overnight. Water and DCM were added and the product was extracted until the aqueous layer stayed colourless. The organic fractions were then washed several times with diluted HCl (couple of drops of 10% HCl in water), dried with magnesium sulfate and the solvent was removed under reduced pressure. The crude was then

purified by flash chromatography (DCM – DCM/MeOH/TEA 50:50: 4 drops/ 10mL) to afford **DPP-R** as a red solid (17.8 mg, 31%). **Mp:** 170-173 °C. The ^{1}H NMR spectrum of **DPP-Ru** in deuterated dichloromethane (DCM) results in a high number of unresolved signals, presumably due to self-aggregation. Addition of TEA lead to a well-resolved ^{1}H NMR spectrum (Figure S5), attributed to the deprotonation of the carboxylic acid, but does not allow for full interpretration of the aliphatic region. **^{1}H NMR (500 MHz, methylene chloride-d$_2$) δ**: 8.19 – 8.15 (m, 3H), 8.04 (d, *J* = 7.6 Hz, 2H), 7.91 – 7.84 (m, 6H), 7.75 (d, *J* = 8.5 Hz, 2H), 7.71 – 7.63 (m, 6H), 7.61 (d, *J* = 6.7 Hz, 2H), 7.57 (d, *J* = 8.2 Hz, 2H), 7.52 (d, 1H), 7.21 (d, *J* = 8.3 Hz, 2H), 6.92 – 6.87 (m, 4H), 3.80 (s, 4H), 2.86 (s, 4H), 2.23 (s, 3H). **^{13}C NMR (126 MHz, methylene chloride-d$_2$) δ:** 172.6, 162.6, 159.7, 157.9, 151.4, 151.4, 151.1, 148.4, 148.3, 147.12, 143.1, 140.9, 140.4, 137.8, 135.6, 135.0, 129.9, 129.4, 129.2, 128.9, 128.6, 127.4, 127.1, 127.0, 126.1, 125.6, 125.4, 124.9, 124.8, 123.3, 110.3, 110.0, 44.9, 38.6, 36.3, 35.5, 31.9, 29.7, 29.6, 29.3, 28.2, 23.7, 23.7, 22.8, 22.8, 22.7, 20.5, 13.9, 13.7, 13.7, 10.2, 10.8, 7.8. **UV-vis (DMF) λ_{max}/nm (ε × 10^{-3} /M^{-1} cm^{-1}):** 302(39.32), 330(23.64), 375(sh, 33.72), 390(35.52), 498(30.70). **FT-IR (ATR) σ/cm^{-1}:** 2955, 2923, 2852, 1709, 1669, 1617, 1593, 1497, 1457, 1439, 1365, 1310, 1261, 1234, 1091, 1034, 1019, 1005, 811, 770. **HRMS (ESI/FTMS) m/z**: $[M + H]^+$ Calcd for $C_{73}H_{72}O_8N_7{}^{102}Ru_1{}^{32}S_1$ 1308.4201; Found 1308.4206.

Supporting figures

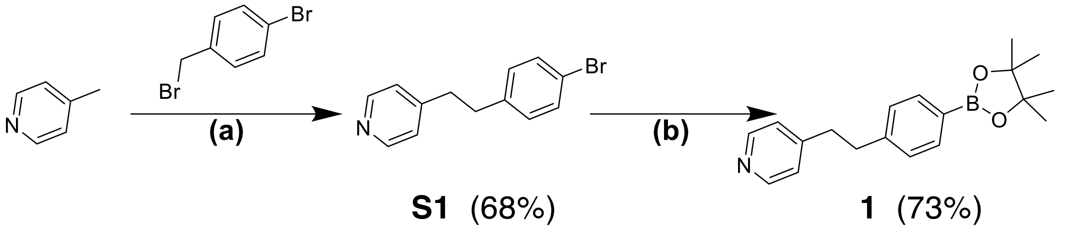

Scheme S1. Synthesis of **1**. Conditions: (a) LDA, THF, N_2, −78 °C, 12 h; (b) dry 1,4-dioxane, [$PdCl_2$(dppf)], KOAc, B_2pin_2, 80 °C, 6 h**.**

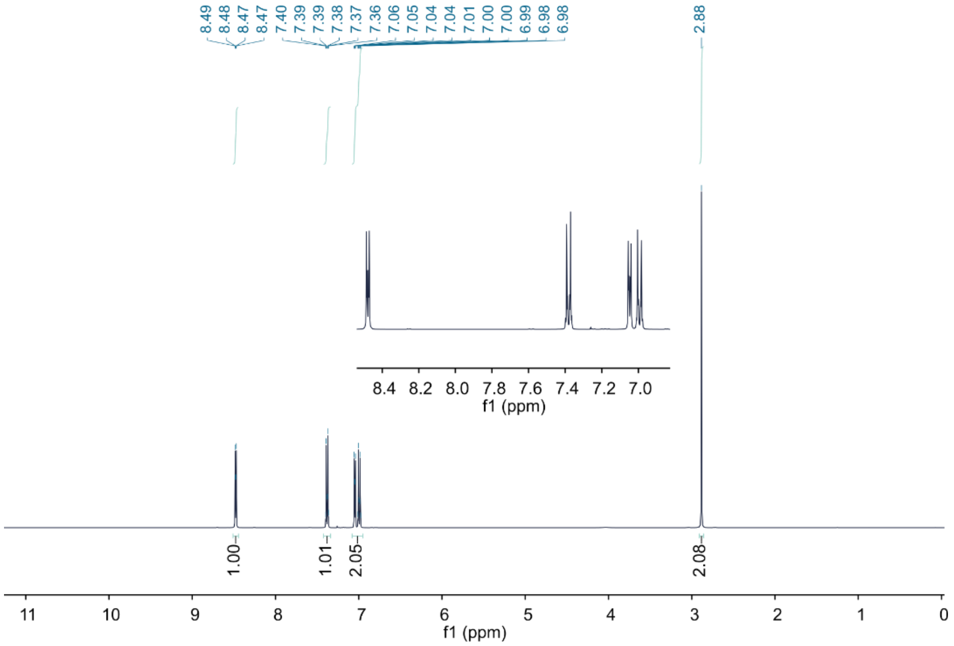

Figure S1. ^{1}H NMR (400 MHz, chloroform-*d*) spectrum of **S1**.

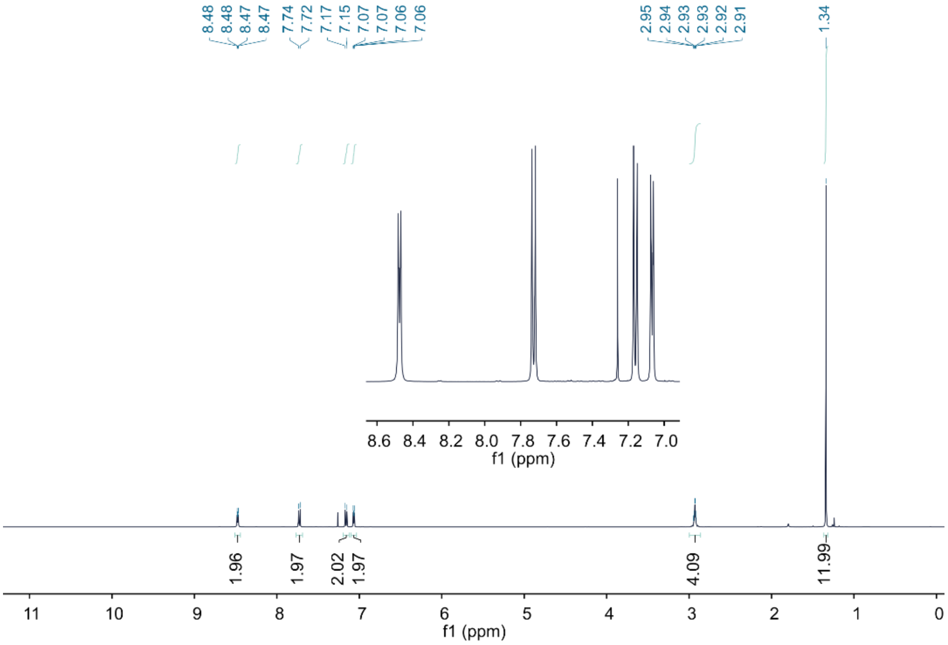

Figure S2. ^{1}H NMR (400 MHz, chloroform-*d*) spectrum of **1**.

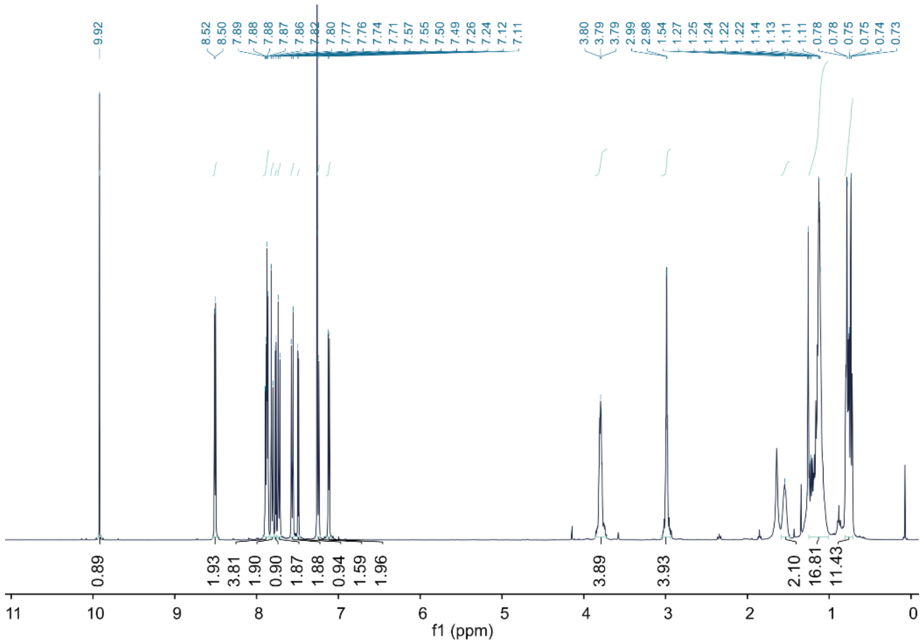

Figure S3. ^{1}H NMR (400 MHz, chloroform-*d*) spectrum of $\mathbf{DPP_{II}}$.

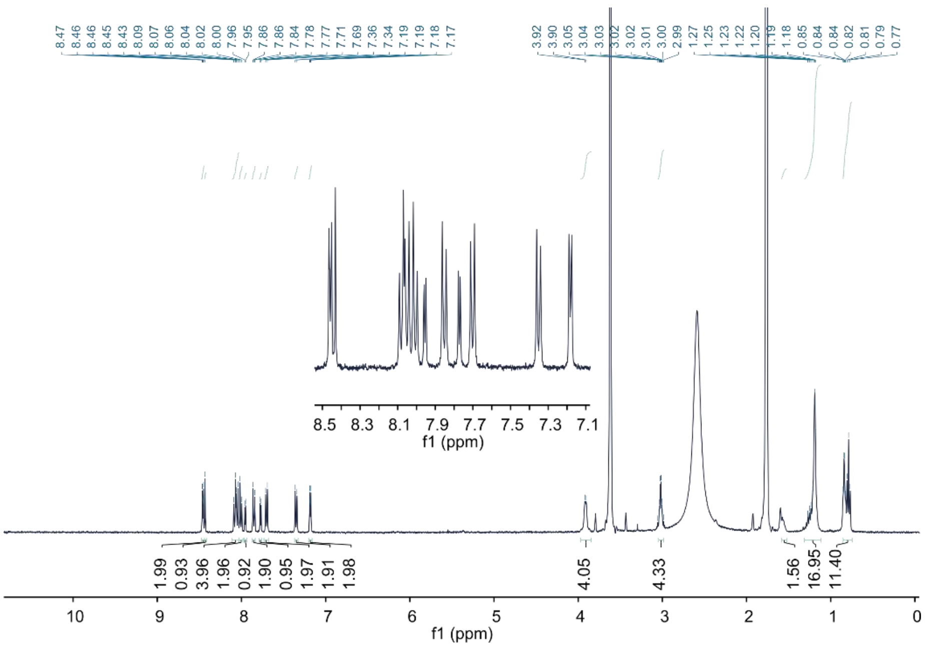

Figure S4. ^{1}H NMR (400 MHz, THF-d_8) spectrum of **DPP$_{dye}$**.

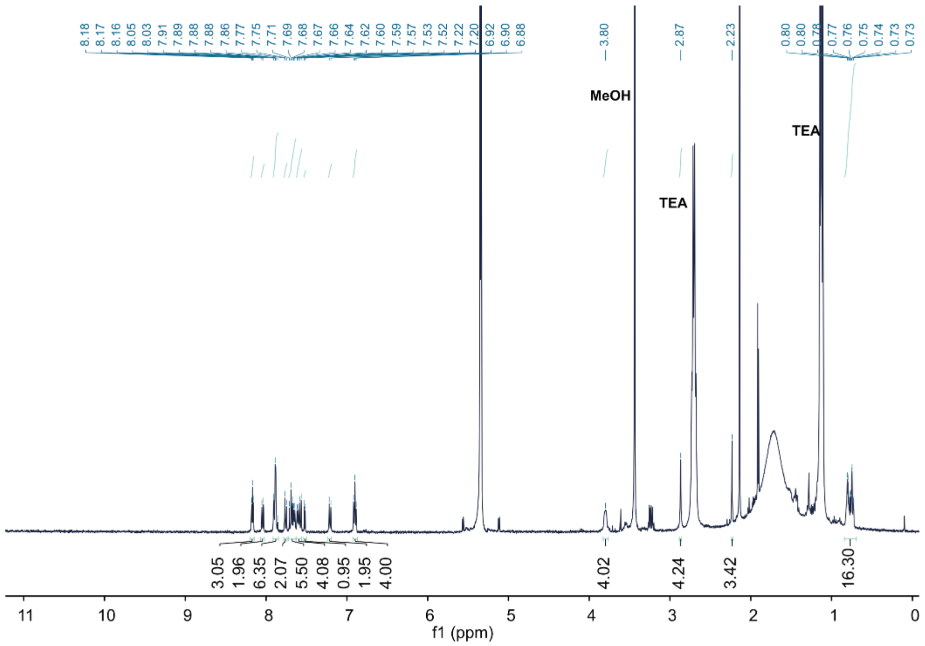

Figure S5. ^{1}H NMR (500 MHz, methylene chloride-d_2) spectrum of **DPP-Ru**.

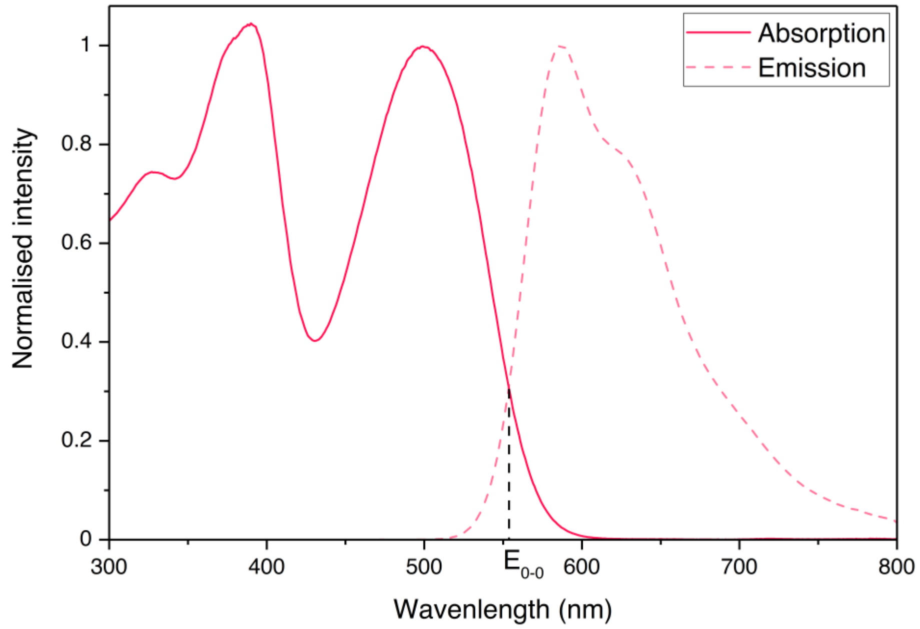

Figure S6. Normalised UV-vis absorption and emission spectra of $\mathbf{DPP_{dye}}$ in DMF solution at room temperature, highlighting the position of $E_{0\text{-}0}$.

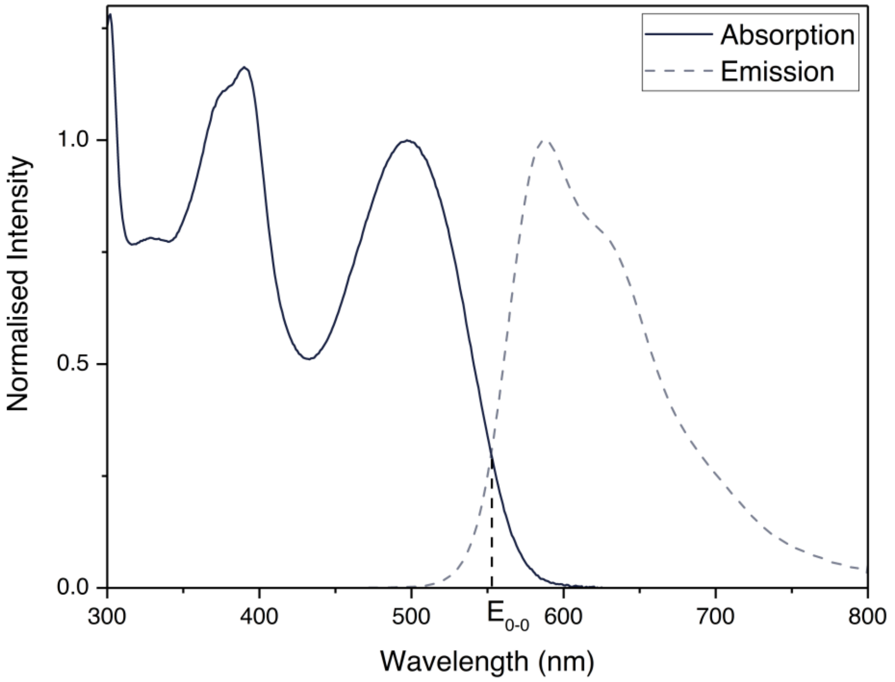

Figure S7. Normalised UV-vis absorption and emission spectra of **DPP-Ru** in DMF solution at room temperature, highlighting the position of $E_{0\text{-}0}$.

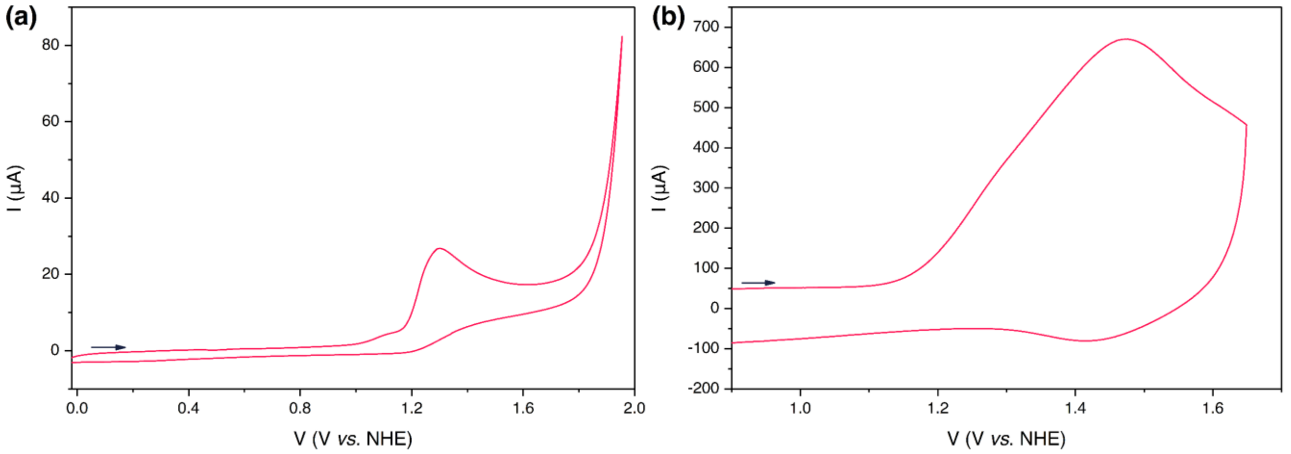

Figure S8. (a) Cyclic voltammogram of **DPP_{dye}** in DMF solution containing $TBABF_4$ (0.1 M, $TBABF_4$ = tetrabutylammonium tetrafluoroborate) as a supporting electrolyte at room temperature in a N_2 atmosphere. Scan rate = 100 mV s^{-1}. (b) Cyclic voltammogram of a mITO|**DPP_{dye}** electrode in MeCN solution containing $TBABF_4$ (0.1 M, $TBABF_4$ = tetrabutylammonium tetrafluoroborate) as a supporting electrolyte at room temperature in a N_2 atmosphere. Scan rate = 50 mV s^{-1}.

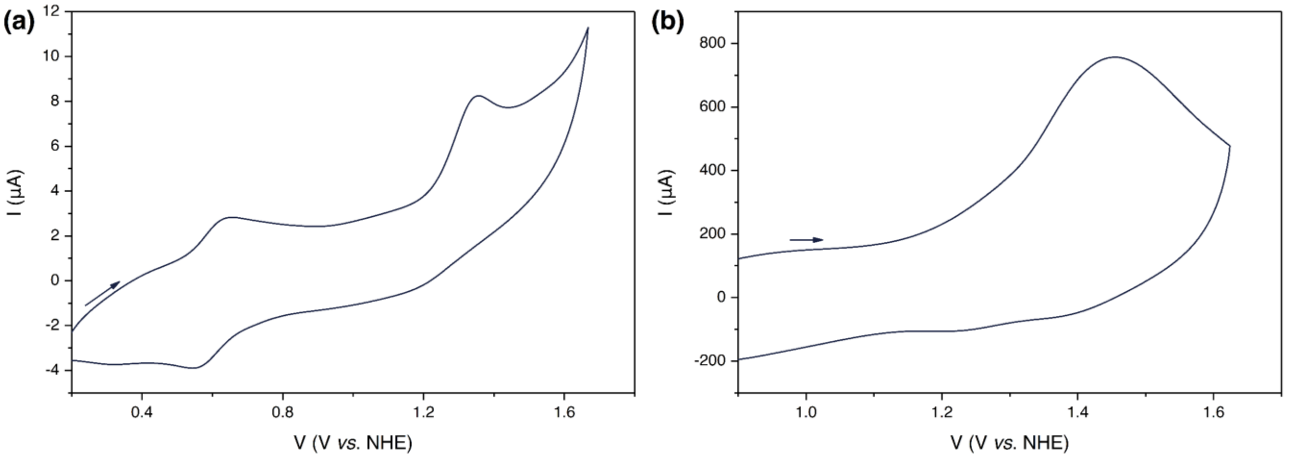

Figure S9. (a) Cyclic voltammogram of **DPP-Ru** in DMF solution containing $TBABF_4$ (0.1 M, $TBABF_4$ = tetrabutylammonium tetrafluoroborate) as a supporting electrolyte at room temperature in a N_2 atmosphere. Scan rate = 50 mV s^{-1}. (b) Cyclic voltammogram of a mITO|**DPP-Ru** electrode in MeCN solution containing $TBABF_4$ (0.1 M, $TBABF_4$ = tetrabutylammonium tetrafluoroborate) as a supporting electrolyte at room temperature in a N_2 atmosphere. Scan rate = 50 mV s^{-1}.

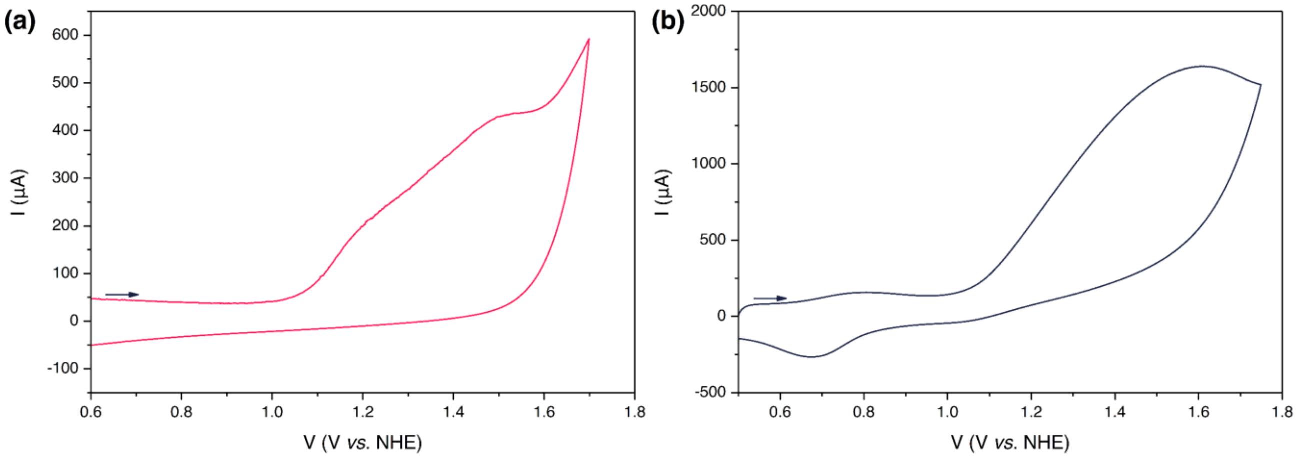

Figure S10. (a) Cyclic voltammogram of a mITO|**DPP$_{dye}$** electrode in NaOAc buffer (0.1 M, pH 5.6) at room temperature in a N_2 atmosphere. Scan rate = 50 mV s^{-1}. (b) Cyclic voltammogram of a mITO|**DPP-Ru** electrode in NaOAc buffer (0.1 M, pH 5.6) at room temperature in a N_2 atmosphere. Scan rate = 50 mV s^{-1}.

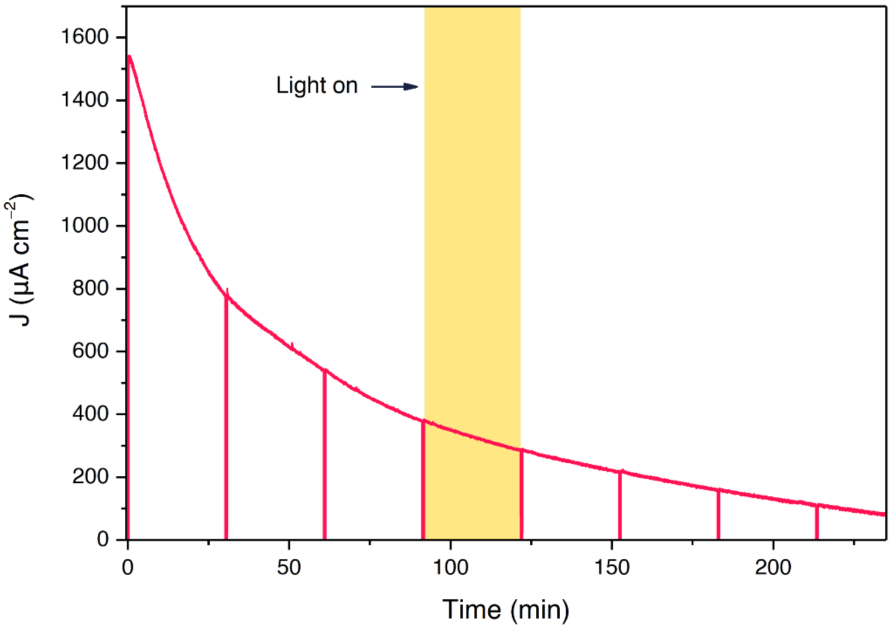

Figure S11. Chronoamperometry results of mTiO$_2$|**DPP$_{dye}$** electrode at +0.2 V *vs.* NHE under chopped light illumination. Conditions: aquoeus TEOA (0.1 M, pH 7), 100 mW cm^{-2}, AM 1.5G, $\lambda >$ 420 nm, N_2 purged, room temperature.

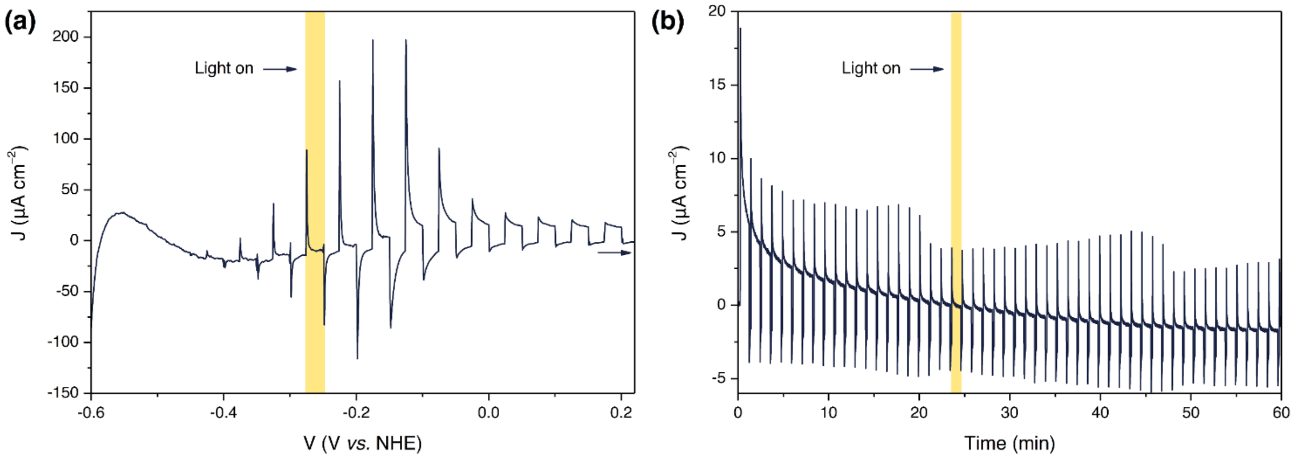

Figure S12. Photoelectrochemistry of $mTiO_2$|**DPP-Ru** electrode. Conditions: NaOAc buffer (0.1 M, pH 5.6), 100 mW cm^{-2}, AM 1.5G, $\lambda > 420$ nm, N_2 purged, room temperature. (a) Linear sweep voltammogram with chopped illumination (v = 5 mV s^{-1}). (b) Chronoamperometry results at +0.2 V *vs.* NHE under chopped light illumination.

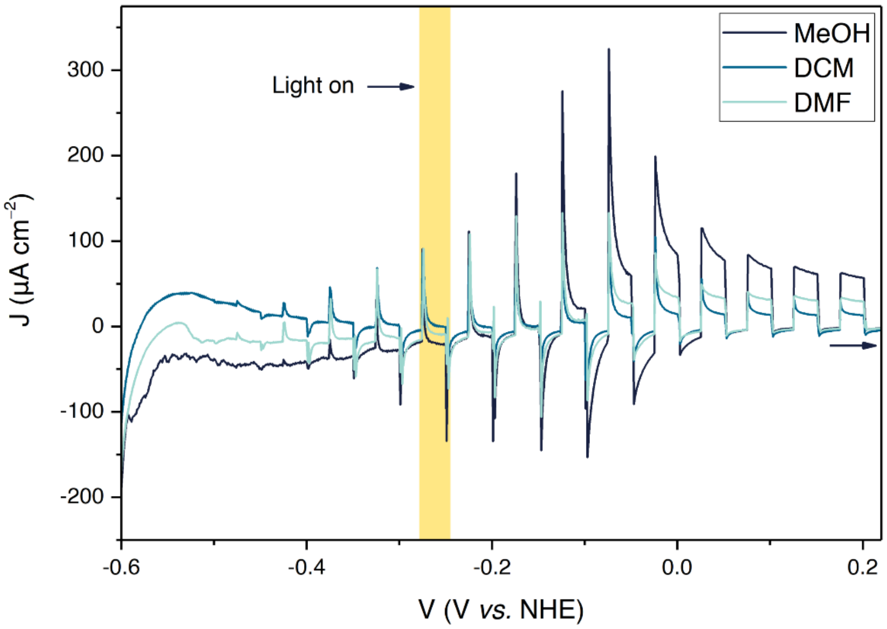

Figure S13. Linear sweep voltammogram with chopped illumination (v = 5 mV s^{-1}) of $TiCl_4$-$mTiO_2$|**DPP-Ru** electrodes using different solvents for the immobilisation bath. Conditions: NaOAc buffer (0.1 M, pH 5.6), 100 mW cm^{-2}, AM 1.5G, $\lambda > 420$ nm, N_2 purged, room temperature.

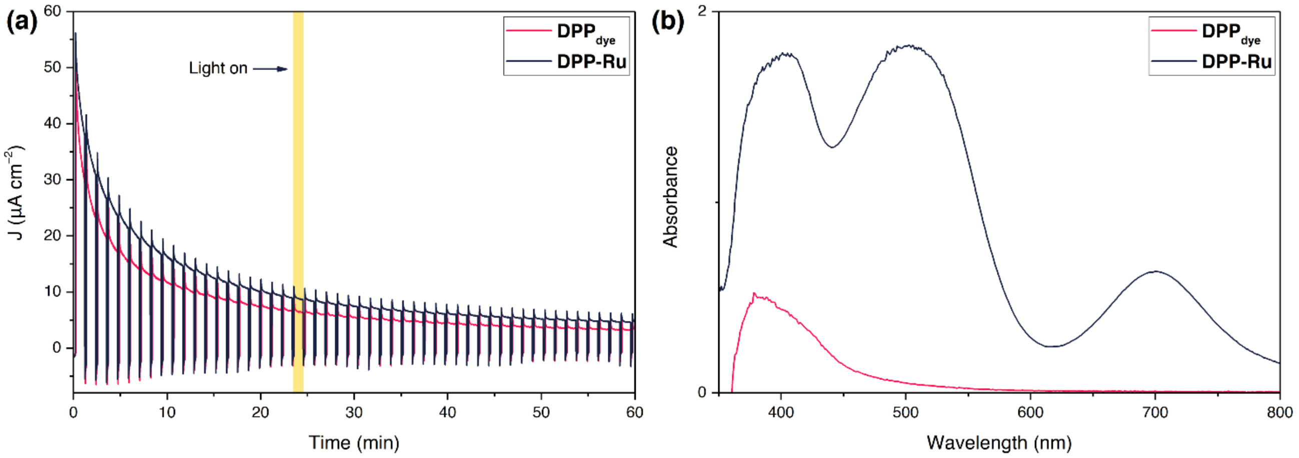

Figure S14. (a) Chronoamperometry results at +0.2 V *vs.* NHE under chopped light illumination of $TiCl_4$-mTiO_2|$\mathbf{DPP_{dye}}$ and $TiCl_4$-mTiO_2|**DPP-Ru** electrodes. Conditions: NaOAc buffer (0.1 M, pH 5.6), 100 mW cm^{-2}, AM 1.5G, $\lambda > 420$ nm, N_2 purged, room temperature. (b) UV-vis absorption spectra of the electrodes after the chronoamperometry experiment. The peak at 700 nm is attributed to the formation of oxidised ruthenium species.[7]

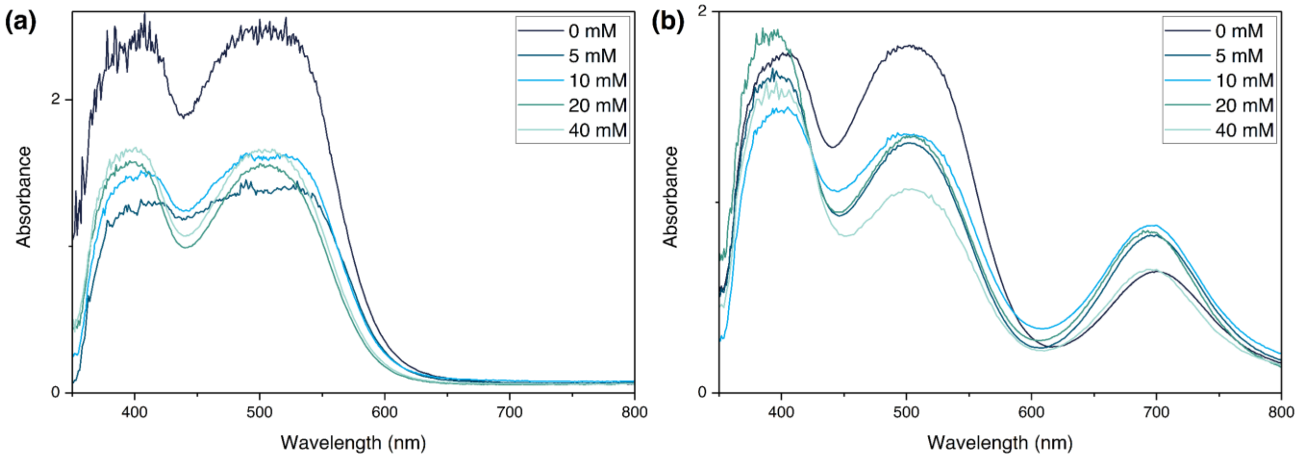

Figure S15. UV-vis absorption spectra of $TiCl_4$-mTiO_2|**DPP-Ru** electrodes prepared by immobilisation in a bath containing **DPP-Ru** (0.1 mM) and CDCA (varying concentrations) in MeOH. (a) Fresh electrodes. As expected, there is a decrease in the absorbance when CDCA is added. However, there is no significant decrease in the absorption from **DPP-Ru** upon increasing the concentration of CDCA from 5 to 40 mM. (b) Electrodes after the photoelectrochemical experiments. The peak at 700 nm is attributed to the formation of oxidised ruthenium species.[7]

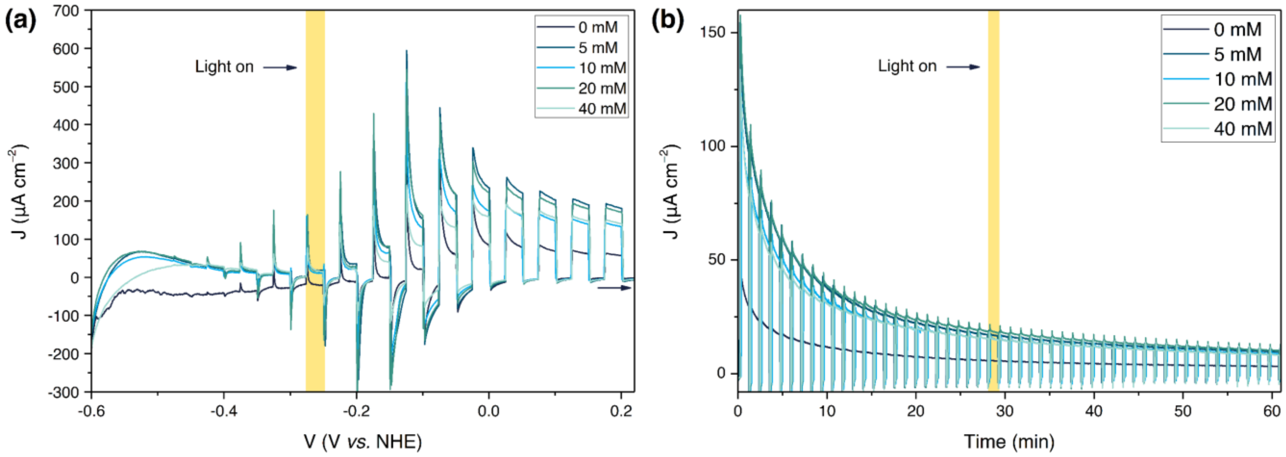

Figure S16. Photoelectrochemistry of mTiO$_2$|**DPP-Ru** electrodes prepared by immobilisation in a bath containing **DPP-Ru** (0.1 mM) and CDCA (varying concentrations) in MeOH. Conditions: NaOAc buffer (0.1 M, pH 5.6), 100 mW cm^{-2}, AM 1.5G, λ > 420 nm, N_2 purged, room temperature. (a) Linear sweep voltammogram with chopped illumination (v = 5 mV s^{-1}). (b) Chronoamperometry results at +0.2 V *vs.* NHE under chopped light illumination.

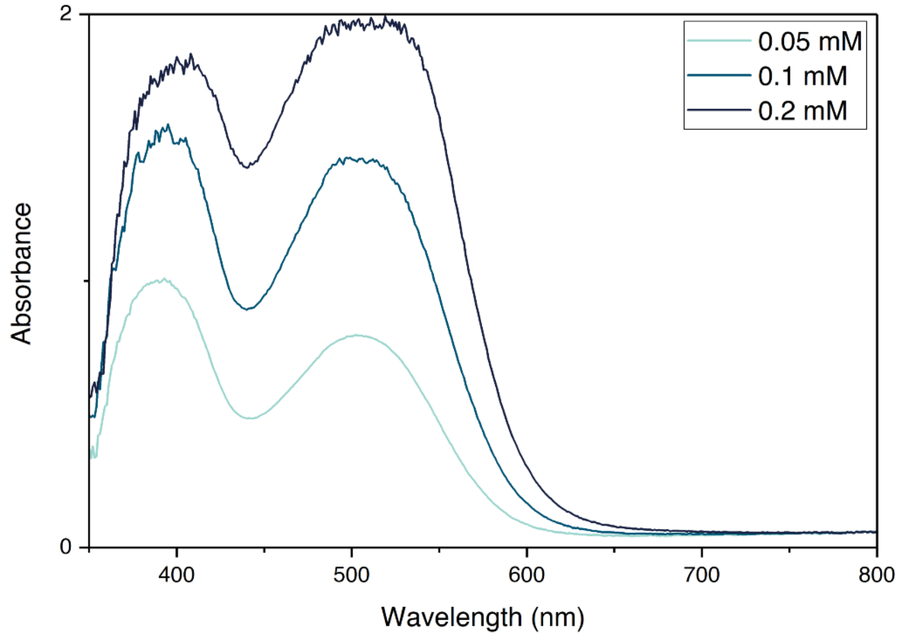

Figure S17. UV-vis absorption spectra of fresh $TiCl_4$-$mTiO_2$|**DPP-Ru** electrodes prepared by immobilisation in a bath containing **DPP-Ru** (varying concentrations) and CDCA (20 mM) in MeOH.

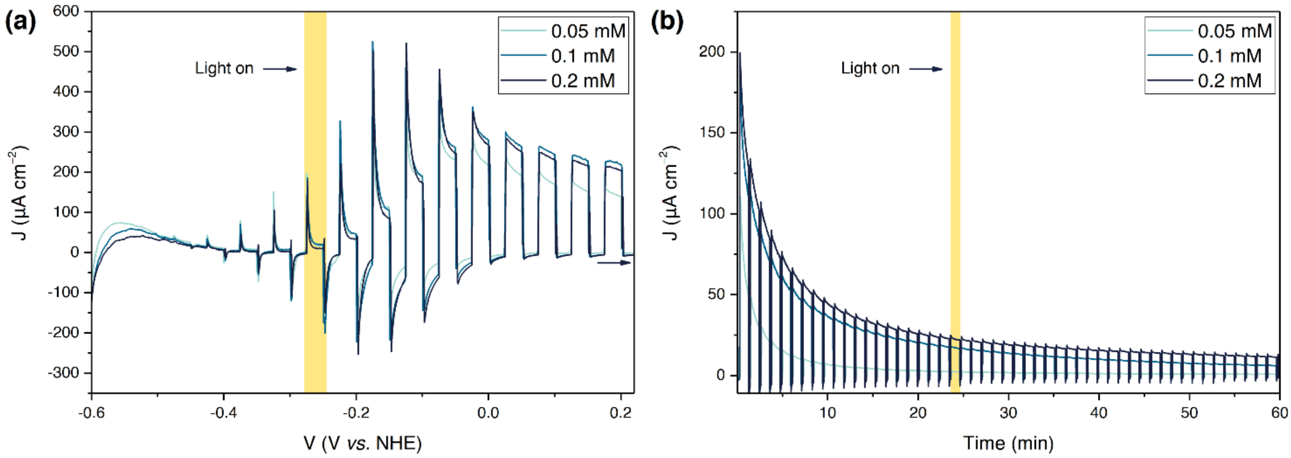

Figure S18. Photoelectrochemistry of $mTiO_2$|**DPP-Ru** electrodes prepared by immobilisation in a bath containing **DPP-Ru** (varying concentrations) and CDCA (20 mM) in MeOH. Conditions: NaOAc buffer (0.1 M, pH 5.6), 100 mW cm^{-2}, AM 1.5G, λ > 420 nm, N_2 purged, room temperature. (a) Linear sweep voltammogram with chopped illumination (v = 5 mV s^{-1}). (b) Chronoamperometry results at +0.2 V *vs.* NHE under chopped light illumination.

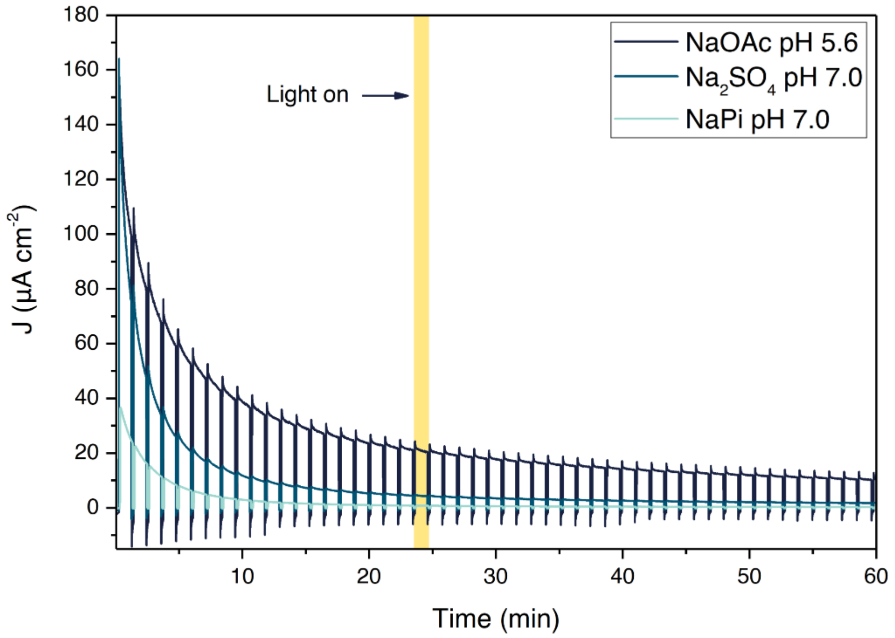

Figure S19. Chronoamperometry results at +0.2 V *vs.* NHE under chopped light illumination of $TiCl_4$-mTiO_2|**DPP-Ru**/CDCA electrodes prepared by immobilisation in a **DPP-Ru** (0.1 mM) and CDCA (20 mM) bath in MeOH. Conditions: NaOAc buffer (0.1 M, pH 5.6), sodium sulfate electrolyte (Na_2SO_4, 0.1 M, pH 7.0) or sodium phosphate buffer (NaPi, 0.1 M, pH 7.0). 100 mW cm^{-2}, AM 1.5G, $\lambda > 420$ nm, N_2 purged, room temperature.

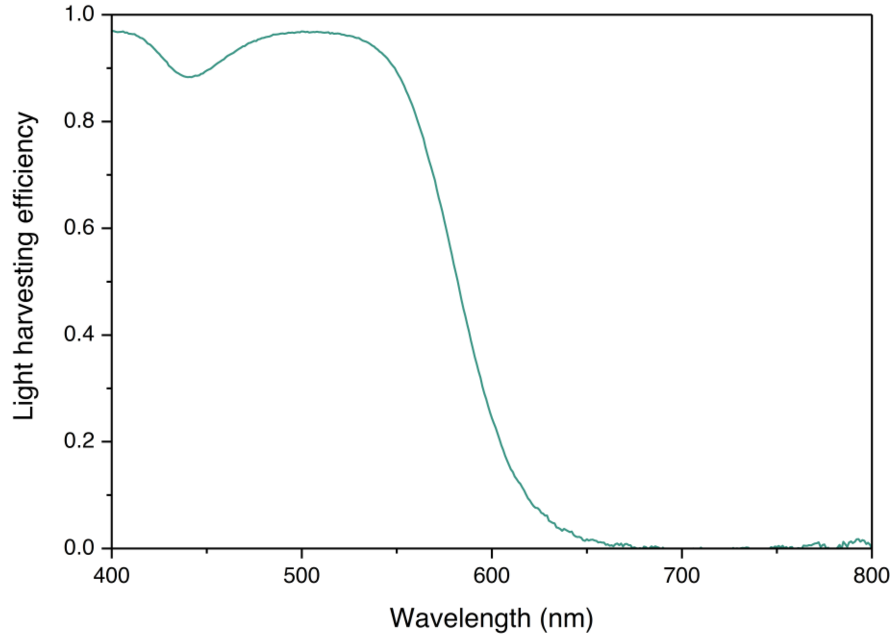

Figure S20. Light harvesting efficiency of a $TiCl_4$-mTiO_2|**DPP-Ru**/CDCA electrode prepared by immobilisation in a **DPP-Ru** (0.1 mM) and CDCA (20 mM) bath in MeOH. The light harvesting efficiency was calculated ($1 - 10^{-absorbance}$) after correcting the absorbance at 800 nm to 0.

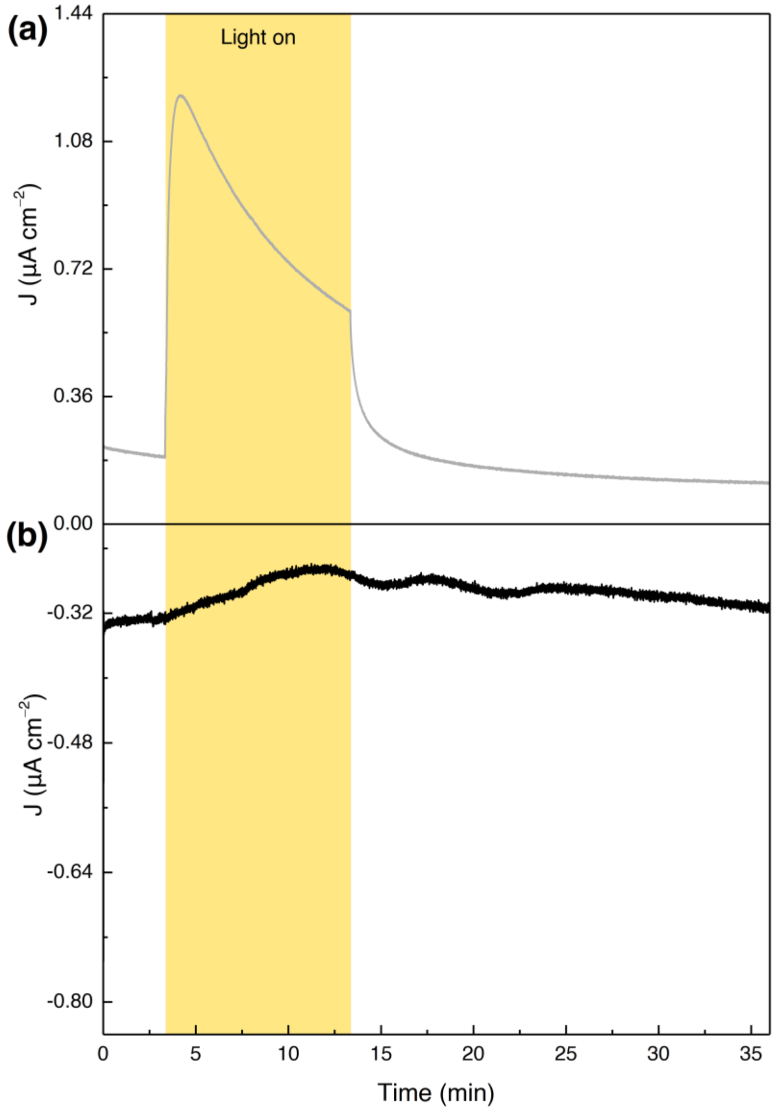

Figure S21. Collector-generator experiment for a $TiCl_4$-mTiO_2 electrode. Conditions: NaOAc (0.1 M, pH 5.6) with $NaClO_4$ (0.4 M), 100 mW cm^{-2}, AM 1.5G, λ > 420 nm, N_2 purged, room temperature. (a) photocurrent trace of the generator with an applied potential of +0.2 *vs.* NHE. (b) current trace of the collector with an applied potential of −0.6 *vs.* NHE.

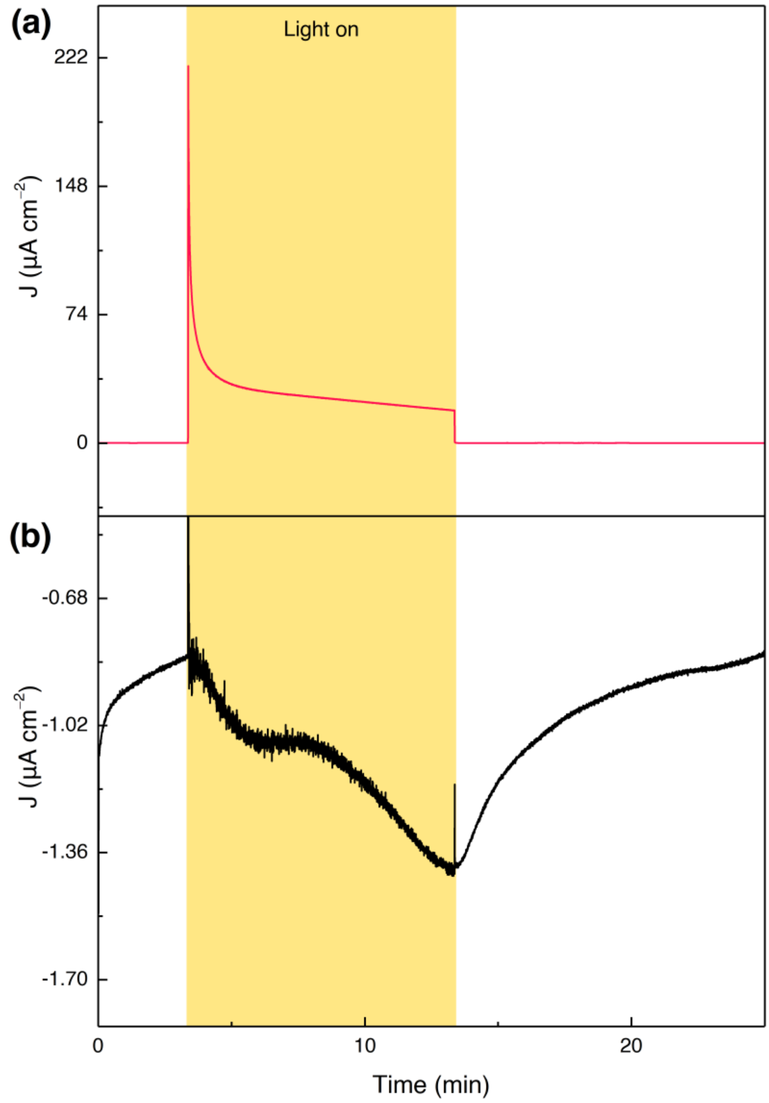

Figure S22. Collector-generator experiment for a $TiCl_4$-mTiO_2|**DPP_{dye}** electrode. Conditions: NaOAc (0.1 M, pH 5.6) with $NaClO_4$ (0.4 M), 100 mW cm^{-2}, AM 1.5G, $\lambda > 420$ nm, N_2, room temperature. (a) photocurrent trace of the generator with an applied potential of +0.2 *vs.* NHE. (b) current trace of the collector with an applied potential of −0.6 *vs.* NHE.

Supporting references

1 J. J. Leung, J. Warnan, K. H. Ly, N. Heidary, D. H. Nam, M. F. Kuehnel and E. Reisner, *Nat. Catal.*, 2019, **2**, 354–365.

2 N. Elgrishi, M. B. Chambers, X. Wang and M. Fontecave, *Chem. Soc. Rev.*, 2017, **46**, 761–796.

3 B. D. Sherman, M. V Sheridan, C. J. Dares and T. J. Meyer, *Anal. Chem.*, 2016, **88**, 7076–7082.

4 J. Warnan, L. Favereau, Y. Pellegrin, E. Blart, D. Jacquemin and F. Odobel, *J. Photochem. Photobiol. A Chem.*, 2011, **226**, 9–15.

5 Y. Gao, X. Ding, J. Liu, L. Wang, Z. Lu, L. Li and L. Sun, *J. Am. Chem. Soc.*, 2013, **135**, 4219–4222.

6 L. Duan, L. Wang, A. K. Inge, A. Fischer, X. Zou and L. Sun, *Inorg. Chem.*, 2013, **52**, 7844–7852.

7 J. J. Concepcion, D. K. Zhong, D. J. Szalda, J. T. Muckerman and E. Fujita, *Chem. Commun.*, 2015, **51**, 4105–4108.

End of ESI
